# Membrane stiffening by STOML3 facilitates mechanosensation in sensory neurons

**DOI:** 10.1038/ncomms9512

**Published:** 2015-10-07

**Authors:** Yanmei Qi, Laura Andolfi, Flavia Frattini, Florian Mayer, Marco Lazzarino, Jing Hu

**Affiliations:** 1 Sensory Mechanotransduction, Centre for Integrative Neuroscience, Otfried-Mueller-Strasse 25, 72076 Tuebingen, Germany; 2 Istituto Officina dei Materiali Consiglio Nazionale delle Ricerche, Laboratorio TASC, 34149 Basovizza, Trieste, Italy

## Abstract

Sensing force is crucial to maintain the viability of all living cells. Despite its fundamental importance, how force is sensed at the molecular level remains largely unknown. Here we show that stomatin-like protein-3 (STOML3) controls membrane mechanics by binding cholesterol and thus facilitates force transfer and tunes the sensitivity of mechano-gated channels, including Piezo channels. STOML3 is detected in cholesterol-rich lipid rafts. In mouse sensory neurons, depletion of cholesterol and deficiency of STOML3 similarly and interdependently attenuate mechanosensitivity while modulating membrane mechanics. In heterologous systems, intact STOML3 is required to maintain membrane mechanics to sensitize Piezo1 and Piezo2 channels. In C57BL/6N, but not *STOML3*^−/−^ mice, tactile allodynia is attenuated by cholesterol depletion, suggesting that membrane stiffening by STOML3 is essential for mechanical sensitivity. Targeting the STOML3–cholesterol association might offer an alternative strategy for control of chronic pain.

## Abstract (web summary)

To maintain viability, cells must be able to sense and respond to mechanical stimuli. Here, Qi *et al*. show that the STOML3 protein acts in mechanosensation by binding cholesterol and regulating membrane stiffness which can in turn regulate ion flux through mechanosensitive channels.

## Graphical abstract

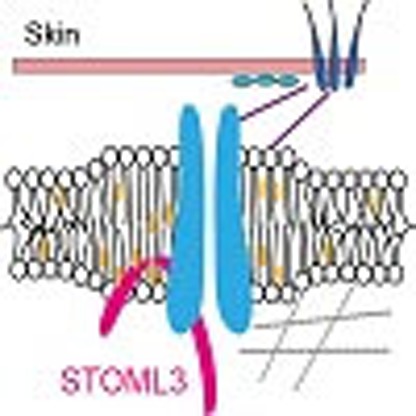

Across the entire biosphere, sensing of mechanical force appears to be critical for all living organisms to interact with the internal and external physical environment. Despite its fundamental importance for survival, how mechanical force is sensed at the molecular level in mammalian sensory neurons remains largely unknown. Most of our knowledge about the molecular entity of mechanosensation have emerged from studying the nematode *Caenorhabditis elegans*^1^. Among mammalian *mec*-homologues, so far, only one protein—a homologue of MEC-2, the stomatin-like protein-3 (STOML3)–has been shown to be essential for touch sensation in mouse^2^. However, the molecular mechanisms by which STOML3 contributes to mechanotransduction remain mysterious. Intriguingly, in heterologous systems, STOML3 has been recently reported to tune the sensitivity of newly discovered mechanically gated Piezo channels^3^, confirming the involvement of STOML3 in mechanotransduction. Yet, little is known about how this works.

Using whole-cell patch-clamp techniques, three distinctive types of mechanosensitive currents can be recorded from cultured dorsal root ganglion (DRG) neurons. According to the inactivation time constant *τ*_1_, they can be classified as rapidly adapting (RA) (τ_1_<5 ms), intermediately adapting (IA) (τ_1_<50 ms) and slowly adapting (SA) (no adaptation during a 500-ms stimulus)^2^,^3^,^4^,^5^,^6^,^7^. The two most common current types, RA- and SA-mechanosensitive currents, represent two distinct channel gating mechanisms: tether model and membrane force model, respectively^1^,^8^. The function of RA channels depends on the presence of a protein tether that may directly or indirectly couple the mechanosensitive channel to a laminin-containing matrix for transferring mechanical force. This coupling could be suppressed by Laminin 332. On the contrary, SA channels do not require a tether protein to accomplish its mechanosensitivity, suggesting SA channels might be directly gated by membrane stretch^4^,^5^,^6^,^8^. However, in *STOML3*^−/−^ mice, the proportions of neurons expressing RA- and SA-mechanosensitive currents decrease conjointly^2^,^3^, indicating that STOML3 may be involved in a more general mechanism independent of gating models in modulating the function of mechanosensitive channels.

Regardless of the gating mechanisms, all mechanosensitive ion channels, including Piezo channels, are embedded in a membrane bilayer and subjected to the mechanical properties of surrounding lipids. Altering the internal membrane force profile has been suggested to not only operate the sensitivity of stretch-activated channels, but also affect the activity of channels gated by force delivered through a tether protein^9^,^10^. Lipid rafts, known as dynamic nanoscale microdomains enriched in cholesterol, sphingolipid and other specific proteins, modify the physical properties of their neighbouring membrane such as thickness, stiffness and pressure profile^11^. For instance, disruption of lipid raft integrity by cholesterol depletion has been demonstrated to change membrane stiffness and result in suppression of stretch-activated channels^12^,^13^,^14^,^15^,^16^.

Here we hypothesize that STOML3 tunes sensitivity of mechanically gated ion channels by controlling the membrane mechanical properties of sensory neurons through recruiting cholesterol. We show that STOML3 is detected in the cholesterol-rich membrane fractions. Depletion of membrane cholesterol abolishes SA-mechanosensitive currents and reduces the amplitude of RA-mechanosensitive currents in sensory neurons from C57BL/6N, but not *STOML3*^−/−^ mice. Through atomic force spectroscopy (AFS), we discover that sensory neurons from *STOML3*^−/−^ mouse exhibit softer membrane and smaller membrane tension when compared with wild type (WT). A STOML3 mutant deficient in cholesterol binding fails to increase the mechanosensitivity of Piezo1 and Piezo2 channels, while it significantly reduces the membrane stiffness in which the Piezo channels are embedded in. Finally, tactile allodynia could be attenuated by injecting methyl-β-cyclodextrin (MβCD), a cholesterol-chelating agent, in C57BL/6N but not *STOML3*^−/−^ mice. We suggest that STOML3, as a member of the stomatin-like protein family, regulates the activity of mechanosensitive channels, including Piezo channels via recruiting cholesterol and forming stiffened membrane surrounding the mechanosensitive channels, and thus facilitating the force transfer and increasing the availability of channels. Targeting the ability of STOML3 to associate with cholesterol might offer a novel peripheral means of chronic pain control.

## Results

### Cholesterol depletion alters mechanosensitivity in neurons

**Figure 1:**
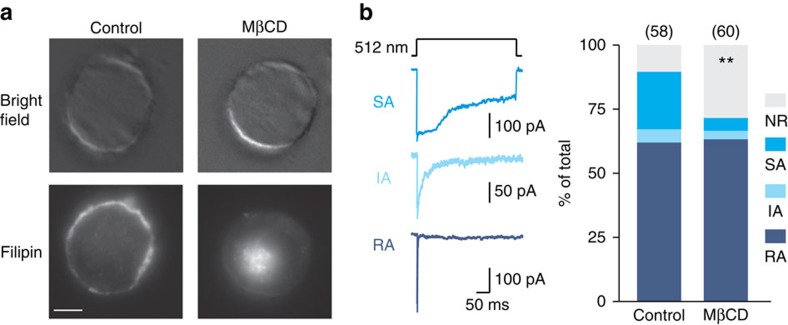
Cholesterol depletion alters mechanosensitivity of sensory neurons. (**a**) Bright field (top) and fluorescence (bottom) images of filipin-labelled neurons treated without (left) or with MβCD (right). Scale bar, 10 μm. (**b**) Left: representative traces of SA-, IA- and RA-mechanosensitive currents evoked by stimulating sensory neuron neurites. Right: stacked histograms showing the proportions of different mechano-gated currents observed in control and MβCD-treated neurons (*χ*^2^-test, *P*<0.01). Note the marked loss of SA-mechanosensitive current in MβCD-treated neurons (Fisher's exact test, *P*<0.01). NR, non-responsive to given displacement 512 nm. **P<0.01.

Mechanical properties of sensory neurons membrane have been recently shown to be essential for the sense of touch in *C. elegans*^17^. In rat sensory neurons, reduced plasma membrane stiffness has been observed after cholesterol depletion, implying that cholesterol-enriched lipid rafts might affect mammalian sensory mechanosensitivity via controlling the membrane force profile in neurons^16^. To investigate whether cholesterol-enriched lipid rafts indeed modulate the sensitivity of mechanically gated ion channels, cultured mouse sensory neurons were treated with or without MβCD (5 mM, 1 h, 37 °C), a commonly utilized agent to efficiently remove membrane cholesterol and to disrupt lipid raft integrity^18^. Treatment with MβCD resulted in a strong decrease of cholesterol in sensory neuron plasma membrane, as indicated by filipin staining, a fluorescent cholesterol-binding molecule (Fig. 1a). Whole-cell patch-clamp recordings were performed immediately after MβCD treatment to record mechanosensitive currents while small mechanical stimuli were given to the neurite (∼512 nm displacement). Remarkably, the proportion of sensory neurons with a SA-mechanosensitive current was significantly reduced from 22% (13/58 cells) in the control to just 5% (3/60 cells) in MβCD-treated cultures (Fisher's exact test, *P*<0.01) (Fig. 1b), while the incidence of RA- or IA-mechanosensitive currents were unaffected (Fig. 1b; Supplementary Table 1). This result demonstrated for the first time a marked influence of the membrane environment on the sensitivity of stretch-activated channels in sensory neurons when gated by force.

**Figure 2:**
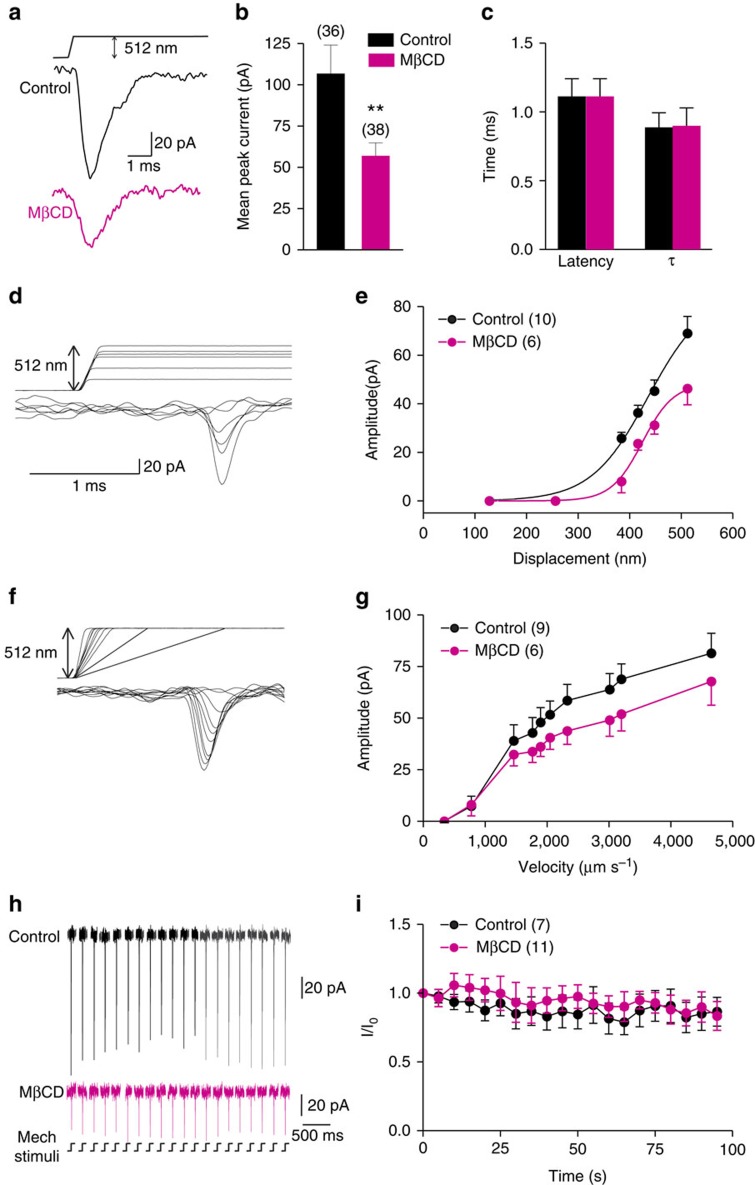
Cholesterol depletion reduces the amplitude of RA currents in sensory neurons. (**a**) Representative traces of RA-mechanosensitive currents recorded in control or MβCD-treated neurons. Quantitative comparison of the peak amplitude (**b**), latency and inactivation time constant (**c**) of the RA currents (unpaired *t*-test, peak amplitude, *P*<0.01; latency, *P*>0.05; inactivation time constant *τ*, *P*>0.05). See also Supplementary Table 1. (**d**) Representative traces of RA currents activated by increasing membrane displacement (from 128 to 512 nm). (**e**) Relationship between displacement and peak current. Data were fitted with Boltzmann distributions (control, *R*^2^=0.82; MβCD, *R*^2^=0.79). See also Supplementary Table 2 and Supplementary Fig. 1. (**f**) Representative traces of RA currents activated by increasing probe velocity (from 341 to 4,654 μm s^−1^). (**g**) Stimulus–response curve of RA currents evoked by increasing probe velocity (two-way ANOVA, *P*<0.01). See also Supplementary Fig. 1. (**h**) Example traces of RA currents evoked by 0.2-Hz 512-nm mechanical stimulus trains. (**i**) No decay in the magnitude of RA currents evoked by a 0.2-Hz mechanical stimulus train in either control or MβCD-treated neurons (time effect: one-way ANOVA, control *P*>0.05, MβCD *P*>0.05; control versus MβCD, two-way ANOVA, *P*>0.05). The number of neurons recorded is indicated in parentheses in each panel. **P<0.01; Error bars indicate s.e.m.

We previously showed that RA channels require a protein tether to transduce mechanical stimulus^5^,^6^. However, it is not clear whether the sensitivity of RA channels can be modulated by their membrane environment. Therefore, we went on to analyse the biophysical properties of RA channels after cholesterol depletion. MβCD treatment significantly reduced the amplitude of RA currents evoked by 512 nm displacement (from 106.8±17.3 pA at −60 mV, *n*=36 to 57.1±7.7 pA, *n*=38; unpaired *t*-test, *P*<0.01; ± indicates s.e.m.; Fig. 2a,b), while having no effect on either the latency or the inactivation time constant (*τ*) of RA currents (Fig. 2c). In both control and MβCD-treated neurons, increased membrane displacement resulted in bigger evoked RA currents (Fig. 2d,e). No shift in the current–displacement curve or change in mechanical threshold (control: 384.0±0.0 nm, *n*=10; MβCD 400.0±7.2 nm, *n*=6; Mann–Whitney test, *P*>0.05; ± indicates s.e.m.) was observed. However, fitting the current versus displacement data to Boltzmann distribution revealed a 45% decrease in the slope sensitivity *s* after MβCD treatment (Fig. 2e; Supplementary Table 2; from 55.37±17.35 pA nm^−1^, *n*=10 to 30.31±10.37 pA nm^−1^, *n*=6; unpaired *t*-test *P*<0.05; ± indicates s.e.m.). The slope sensitivity *s* is proportional to the channel active-state probability^19^,^20^, and the observed reduction suggested that the cholesterol depletion affected RA channel activity. In contrast to the change, we observed in slope sensitivity *s*, the displacement for half-maximal activation (midpoint) *x*_1/2_ was comparable between control and MβCD groups (Supplementary Table 2; 435.8±28.00 nm, *n*=10 and 424.8±11.68 nm, *n*=6, respectively; unpaired *t*-test, *P*>0.05; ± indicates s.e.m.). The kinetics of RA currents was identical for both groups and remained constant regardless of the magnitude of the stimulus (Supplementary Fig. 1). No changes in the midpoint *x*_1/2_ and the kinetics imply that the mechanical gating of RA channels is not affected by cholesterol depletion. It has been shown that RA current is able to encode the dynamic parameters of the mechanical stimulus^21^,^22^. To investigate whether cholesterol depletion alters this property of RA currents, 512 nm mechanical stimuli were applied to the neurite with increasing velocities from 341 to 4,654 μm s^−1^. In control and MβCD-treated neurons, the evoked RA currents were similarly proportional to probe velocity, with increased probe velocity resulting in bigger RA currents (Fig. 2f,g; Supplementary Fig. 1). To test whether RA channels undergo desensitization after MβCD treatment, we applied repetitive stimulus to cultured sensory neurons. In both control and MβCD-treated neurons, repetitive stimulation at an interval of 5 s elicited RA currents with similar amplitude (Fig. 2h,i), indicating that cholesterol depletion did not desensitize RA channels. The ion selectivity of RA channels was not altered by cholesterol depletion either (Supplementary Fig. 1). Altogether, these data revealed that disrupting cholesterol-rich lipid rafts, and thus changing the local internal membrane force profile, had no effect on RA channel kinetics or their gating threshold to mechanical force, implying that RA channels are not directly activated by stretching membrane. This is in agreement with our previous finding that RA channels require a protein tether to transfer the mechanical force^5^,^6^. However, the reduced amplitude of RA currents and the altered slope sensitivity indicate that the number of mechanosensitive channels in an active state may be altered by lipid raft disruption. Taken together, altered membrane mechanics by disruption of lipid raft integrity not only affects the sensitivity of SA channels, but also influences the activity of RA channels.

### Altered membrane mechanics in *STOML3* ^*−/−*^ sensory neurons

**Figure 3:**
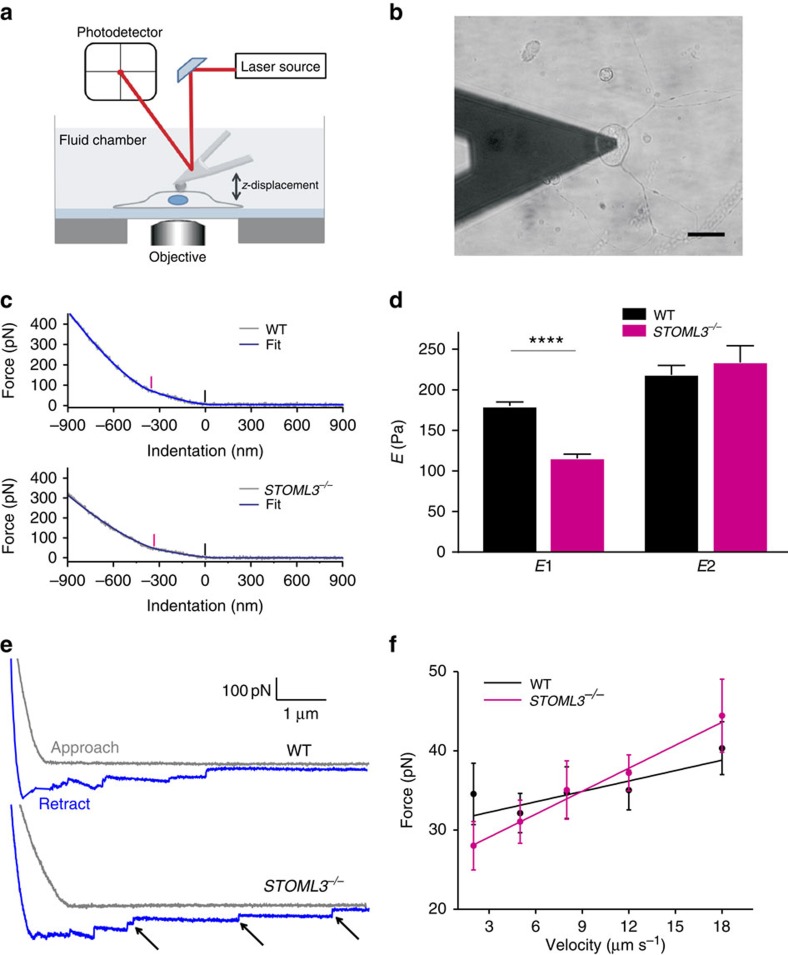
Altered membrane mechanical properties in STOML3-deficient sensory neurons. (**a**) Sketch of AFM set-up used for indentation measurements including a bead (4.5 μm diameter) glued at the end of a tipless cantilever. The cantilever is moved up and down by a piezo system. The bending of the cantilever is measured by a laser beam detected by a photodetector. And the AFM cantilever bending is linearly proportional to the force applied on the cell. (**b**) Differential interference-contrast optical image of the bead cantilever pushed onto a cultured DRG neuron (scale bar, 20 μm). (**c**) Examples of force–indentation curves for WT and *STOML3*^−/−^ sensory neurons as fitted for double contributions: in the curve two regimes with different elastic properties can be identified (black marker, first point for first elastic component *E*_1_, and pink marker second point for second elastic component *E*_2_) (Methods, Supplementary Figs 2 and 3). (**d**) Quantitative comparison of elasticity modulus *E*_1_ and *E*_2_ values obtained by the fitting procedure (WT: *n*=110 neurons from 4 cultures, 4 animals; *STOML3*^−*/*−^: *n*=120 neurons from 4 cultures, 4 animals; two-way ANOVA with *post hoc* Bonferroni's test, *P*<0.05). See also Supplementary Fig. 4. (**e**) Representative force–distance curves (approach in grey, retraction in blue) acquired at 5 μm s^−1^. On the retraction curve, after first detachment force several step-like structures are clearly discernible (indicated by arrows), corresponding to the sequential detachment of membrane tethers from the cantilever. (**f**) Tether force as a function of pulling velocity in WT and *STOML3*^−/−^ sensory neurons (WT: *n*=20 neurons from 2 cultures, 2 animals; *STOML3*^−*/*−^: *n*=19 neurons from 2 cultures, 2 animals). Solid lines are the linear fit of force–velocity curves used to estimate the static tether force at zero *F*_0_ and the effective viscosity *η*_eff_ (Methods, Supplementary Table 2) (*R*-value is 0.6 for WT and 0.97 for *STOML3*^−*/*−^). ****P<0.0001; Error bars indicate s.e.m.

Previously, we have shown that about 35% mechanosensitive channels in sensory neurons do not function without STOML3, regardless of the type of mechanosensitive channels^2^. In view of the fact that both cholesterol depletion and STOML3 deficiency affected the sensitivity of RA and SA channels, we explored the possibility that knocking out STOML3 would also influence the membrane stiffness of sensory neurons. The AFS technique was thus employed to determine the elasticity modulus *E* (that is, stiffness) of sensory neurons from WT and *STOML3*^−/−^ mice. Individual sensory neurons were localized using optical phase-contrast microscopy (Fig. 3a,b). We found that the force required to produce the same indentation on the cell was significantly lower in *STOML3*^−/−^ neurons as compared with WT neurons (Fig. 3c). Living cells can be considered mechanically as a multilayered structure: an external layer including a plasma membrane/cortical actin layer and an internal cytoplasm organization^23^,^24^,^25^,^26^. The stiffness of the external ‘layer' of the sensory neurons was analysed by fitting the force–indentation curves with a variation of the Hertz model that considers the presence of multiple elastic contributions (Fig. 3c; Supplementary Figs 2 and 3; Methods). Using this procedure, we observed that *STOML3*^−/−^ are significantly softer than WT neurons regarding the first elastic component *E*_1_ (Fig. 3d; two-way analysis of variance (ANOVA) with a *post hoc* Bonferroni's multiple comparison test, *P*<0.05), which is mainly related to the mechanics of the plasma membrane and the actin immediately underneath, while for the second elastic component *E*_2_ (internal cell organization), statistically significant difference was not observed (see also Supplementary Fig. 4). Such results suggest that STOML3 acts to selectively increase the stiffness of the external layer of sensory neurons, including plasma membrane and cortical actin.

To further determine whether this decrement in *E*-values at superficial layer of neurons is correlated with membrane mechanical properties, we evaluated membrane viscosity and tension. The force required to pull membrane tethers from neurons at a constant velocity, *V*_t_, was measured and then the dependence of force on velocity was analysed. This procedure allowed us to evaluate the static tether force at zero velocity *F*_0_ and to assess changes in the membrane surface viscosity *η*_eff_ (Fig. 3e,f; Methods). An increase in membrane viscosity in *STOML3*^−/−^ neurons was observed (Supplementary Table 3; 2*πη*_eff_ increased from 0.44±0.16 pN s μm^−1^, *n*=20 to 0.97±0.07 pN s μm^−1^, *n*=19; ± indicates s.e.m.), confirming an alteration of plasma membrane organization by STOML3. The apparent membrane tension, *T*_app_, which is a sum of the membrane–cytoskeleton adhesion energy (*γ*) and in-plane membrane tension (*T*_m_)^27^, was computed from *F*_0_. We found *T*_app_ was lower in *STOML3*^−/−^ than WT sensory neurons (Supplementary Table 3; WT: 42±7% pN μm^−1^, *n*=20; *STOML3*^−/−^: 31±3% pN μm^−1^, *n*=19; ± indicates s.e.m.), suggesting that an intact STOML3 is needed in a sensory neuron to generate a proper membrane tension. Taken together, these results show that STOML3 is critical for maintaining membrane mechanics of sensory neurons and suggest that STOML3, similar to a cholesterol-rich lipid raft, may influence sensory mechanotransduction via stiffening of the neuronal membrane and result in an efficient transfer of force to the mechanically gated ion channels.

### Lipid raft-regulated mechanosensitivity involves STOML3

Given the similarity of cholesterol depletion to STOML3 deficiency, both affected the sensitivity of mechanically gated currents and membrane mechanics. We propose that STOML3 and lipid rafts may regulate the activity of mechanosensitive channels through the same pathway, that is, modulating membrane physical properties.

**Figure 4:**
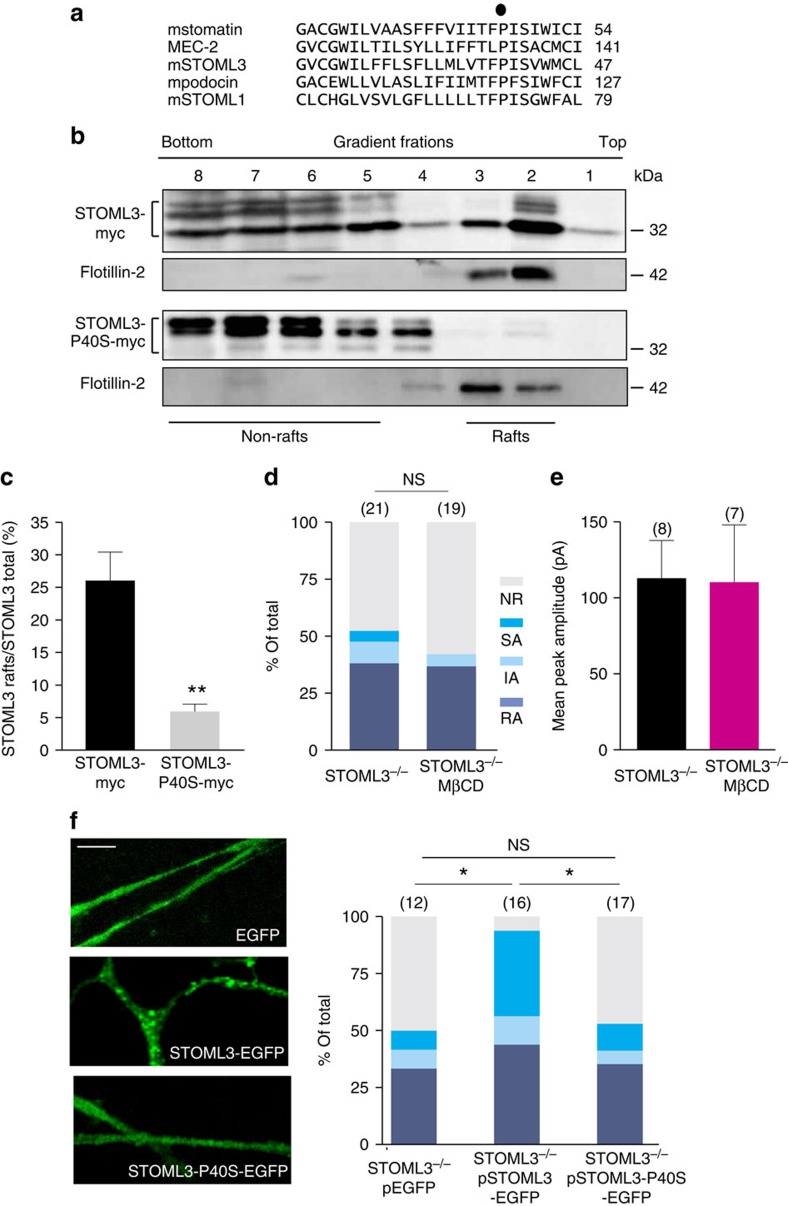
Cholesterol binding is essential for STOML3 to regulate mechanotransduction in sensory neurons. (**a**) Alignment of the deduced amino-acid sequence of mouse STOML3 with its homologues including stomatin, MEC-2 podocin and STOML1. The conserved proline is indicted with a solid circle. (**b**) Representative blots showing that P40S mutation abolishes the distribution of STOML3 in lipid rafts. CHO cells expressing Myc-tagged wild-type STOML3 or P40S mutant were lysed and submitted to sucrose-density-gradient centrifugation. Eight fractions were collected from the top of the gradient (1–8, top to bottom). STOML3 was abundantly detected in both raft fractions (top 2–3) and non-raft fractions (5–8), while STOML3-P40S was mainly detected in non-raft fractions (5–8). STOML3 appeared as a triplet near the predicted molecular weight. The P40S mutation altered the relative intensity of the three bands. See also Supplementary Fig. 5. (**c**) Quantitative comparison of percentage of raft-associated fractions (fractions 1–3) relative to total amount of STOML3 or STOML3-P40 protein (*n*=5 for each, unpaired *t*-test, *P*<0.01). (**d**) Stacked histograms showing the proportions of different mechano-gated currents observed in *STOML3*^−/−^ neurons treated without or with MβCD (*χ*^2^-test, *P*>0.05). MβCD treatment did not significantly reduce the mechanosensitivity of *STOML3*^−/−^ sensory neurons. NS, not significant. (**e**) Quantitative comparison of the peak amplitude of the RA currents recorded from *STOML3*^−/−^ neurons treated without or with MβCD. The amplitude of RA current in *STOML3*^−/−^ neurons was not altered by MβCD treatment (unpaired *t*-test, *P*>0.05). (**f**) Left: representative images of EGFP (top), STOML3-EGFP (middle) and STOML3-P40S-EGFP (bottom) expression in transfected *STOML3*^−/−^ sensory neurons. Note the punctate distribution of STOML3-EGFP along neurites disappeared in *STOML3-P40S-EGFP*-transfected *STOML3*^−/−^ sensory neurons. Scale bar, 10 μm; right: stacked histograms showing the proportions of different mechano-gated currents observed in *STOML3*^−/−^ neurons transfected with *EGFP*, *STOML3-EGFP* or *STOML3-P40S-EGFP* cDNA. Note *STOML3-P40S-EGFP* transfection failed to restore the mechanosensitivity in *STOML3*^−/−^ sensory neurons (*χ*^2^-test, EGFP versus STOML3-EGFP, *P*<0.05; STOML3-EGFP versus STOML3-P40S-EGFP, *P*<0.05; EGFP versus STOML3-P40S-EGFP, *P*>0.05). The number of neurons recorded is indicated in parentheses in each panel. *P<0.05; **P<0.01; Error bars indicate s.e.m.

To test this idea, we first set out to determine whether STOML3 associates with lipid rafts. Many members of the stomatin protein family, including stomatin, STOML1, MEC-2 and podocin, have been found in cholesterol-rich membrane fractions^28^,^29^,^30^,^31^,^32^,^33^,^34^. However, to date there is no direct evidence showing that STOML3 is associated with cholesterol-rich lipid rafts. To examine this, we expressed STOML3 tagged with a myc epitope at its N terminus in Chinese hamster ovarian (CHO) cells and performed sucrose-density-gradient centrifugation of cell lysates. Western blot analysis revealed that STOML3 was enriched in both the fractions towards the top of the gradient and some fractions at the bottom of the gradient, corresponding to the lipid raft fractions and the non-raft membrane fractions, respectively, while the lipid raft marker flotillin-2 was only detected in raft fraction (Fig. 4b; Supplementary Fig. 5). Thus STOML3 binds cholesterol and modulates the physical properties of membrane surrounding its associated mechanosensitive channels.

Next, we treated *STOML3*^−/−^ sensory neurons with MβCD and the mechanically activated response was recorded. Consistent with our previous report^2^, absence of STOML3 significantly reduced the proportion of sensory neurons responding to mechanical stimulation (Supplementary Fig. 6). Intriguingly, cholesterol depletion did not further reduce the mechanosensitivity of *STOML3*^−/−^ neurons. Different from WT sensory neurons, neither the proportion of non-responsive neurons nor the peak amplitude of RA current was altered after MβCD treatment (Fig. 4d,e). Lack of an additive effect of MβCD on mechanosensitive currents in *STOML3*^−/−^ sensory neurons suggests that the regulation of mechanosensitivity by cholesterol-enriched lipid rafts involves STOML3.

### STOML3–cholesterol association regulates mechanosensitivity

Our findings implicate that cholesterol-rich lipid raft controlling mechanosensitivity requires STOML3. To determine whether binding to cholesterol is also essential for the function of STOML3 in regulating the activity of mechanosensitive channels, we sought to specifically disrupt the ability of STOML3 to associate with cholesterol. Mutation of the proline residue in MEC-2 (P134) hydrophobic region has been demonstrated to be able to abolish cholesterol binding^29^ and cause touch insensitivity in *C. elegans*^35^. The proline residue is highly conserved among MEC-2, STOML1, STOML3, stomatin and podocin, indicating a preserved function of this residue for the stomatin protein family^28^,^29^,^36^,^37^ (Fig. 4a). To investigate whether the proline residue is also required for STOML3–cholesterol binding, we substituted proline 40 in STOML3 by a serine residue and examined its raft distribution. STOML3-P40S was mainly detected in the non-raft fractions, confirming that the proline residue is essential for the association between STOML3 and cholesterol-enriched lipid raft (Fig. 4b,c; Supplementary Fig. 5). Next, we transfected *STOML3*^−*/*−^ sensory neurons with WT STOML3 or STOML3-P40S, both tagged with enhanced green fluorescent protein (EGFP) at the C terminus. Consistent with our previous report^2^, fluorescently labelled WT STOML3 was observed in a punctate pattern in neurites, and the expression of WT STOML3 could rescue the deficiency of mechanosensitivity in *STOML3*^−/−^ sensory neurons (15 out of 16 labelled cells exhibited mechanosensitive currents; Fig. 4f). On the contrary, STOML3-P40S was more uniformly distributed, and had no effect on the incidence of mechanosensitive currents (8 out of 17 labelled cells had no mechanosensitive current; Fig. 4f). These results suggest that STOML3 binding cholesterol is crucial for its function in regulating mechanotransduction.

### STOML3 modulates Piezo activity via cholesterol association

**Figure 5:**
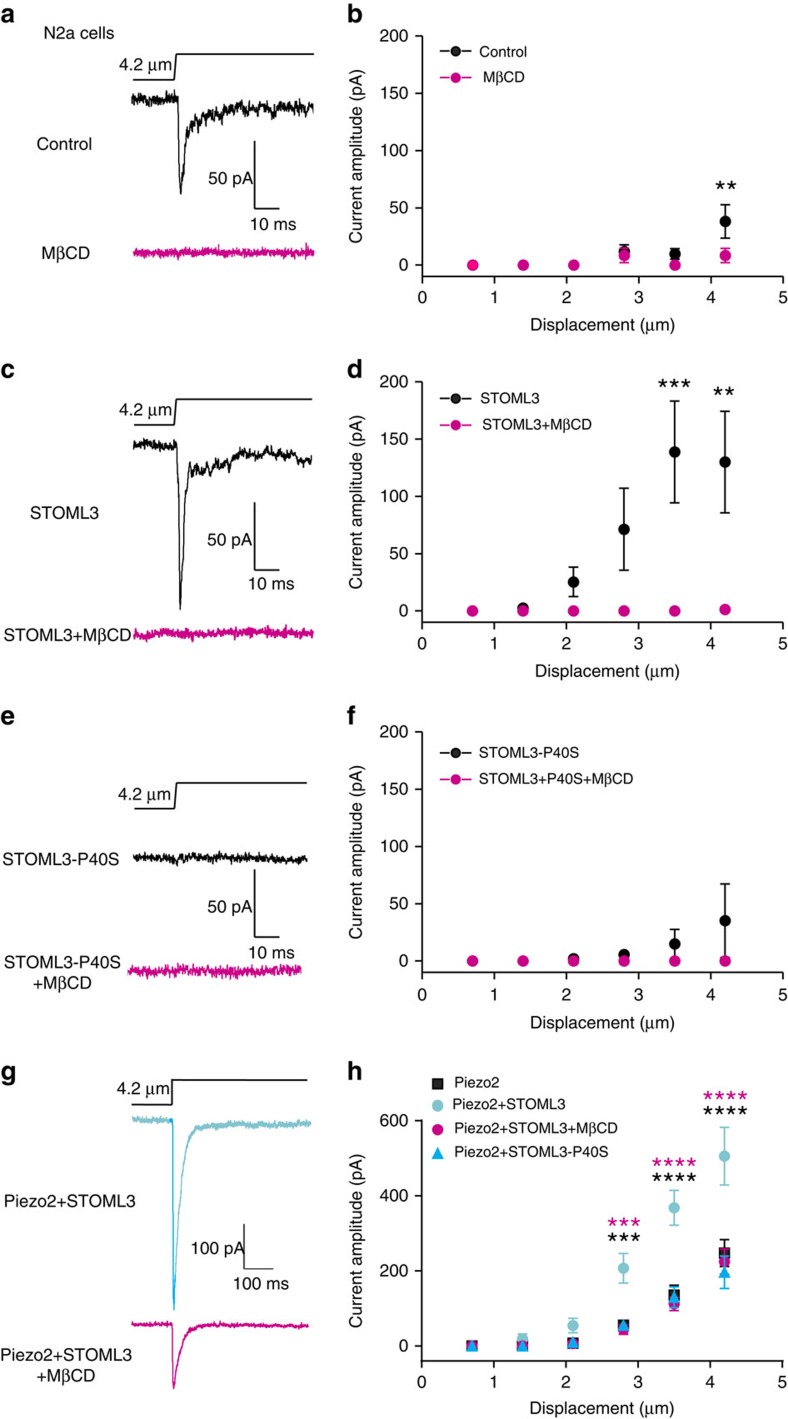
STOML3 modulates Piezo1 and Piezo2 channels activity via association with cholesterol. (**a**,**c**,**e**) Representative traces of mechanically gated currents in N2a cells (**a**) or N2a cells expressing STOML3 (**c**) or N2a cells expressing STOML3-P40S mutant (**e**) treated with or without MβCD at the given indentation (4.2 μm). (**b**) When N2a cells were treated with MβCD (*n*=33), mechanosensitivity was significantly reduced at 4.2 μm displacement compared with cells without MβCD treatment (*n*=27) (two-way ANOVA with *post hoc* Bonferroni's test, *P*>0.05). (**d**) MβCD significantly reduced the mechanosensitive currents in N2a cells overexpressing STOML3 (STOML3: *n*=11, STOML3+MβCD: *n*=8; two-way ANOVA with *post hoc* Bonferroni's test, *P*<0.0001). (**f**) MβCD did not alter the mechanosensitive currents in N2a cells expressing STOML3-P40S mutant (STOML3-P40: *n*=6, STOML3-P40+MβCD: *n*=7; two-way ANOVA with *post hoc* Bonferroni's test, *P*>0.05). (**g**) Representative traces of mechanically gated currents in HEK293 cells expressing Piezo2 and STOML3 treated with or without MβCD at the given indentation (4.2 μm). (**h**) MβCD treatment significantly reduced the potentiation effect of STOML3 on Piezo2-mediated mechanosensitive currents in HEK293 cells, while STOML3-P40S mutant failed to potentiate Piezo2 currents (two-way ANOVA with *post hoc* Bonferroni's test; STOML3 effect: Piezo2 versus Piezo2+STOML3, *P*<0.0001, black *; MβCD effect: Piezo2+STOML3 versus Piezo2+STOML3+MβCD, *P*<0.0001, pink *; STOML3-P40S effect: Piezo2 versus Piezo2+STOML3-P40S, *P*>0.05; Piezo2: *n*=9, Piezo2+STOML3: *n*=7, Piezo2+STOML3+MβCD: *n*=5, Piezo2+STOML3-P40S: *n*=7). **P<0.01, ***P<0.001, ****P<0.0001. Error bars indicate s.e.m.

Our electrophysiological findings demonstrate that cholesterol depletion altered both RA- and SA-mechanosensitive currents in sensory neurons. While at the moment the cation channels that account for SA currents in nociceptors remain unknown, Piezo channels have been recently discovered as true mechanosensitive channels^38^,^39^. And Piezo2 channel mediates RA-mechanosensitive currents in sensory neurons. STOML3 interacts with Piezo channels and sensitizes Piezo1-mediated currents in N2a neuroblastoma cells^3^. We therefore first tested whether STOML3 modulates Piezo1 channel activity through cholesterol-rich lipid rafts. N2a cells, which express low levels of endogenous STOML3, were treated with or without MβCD (5 mM, 1 h, 37 °C). Consistent with previous studies^3^,^40^, mechanosensitive currents with a diversity of inactivation kinetics have been recorded in N2a cells. After MβCD treatment, we have observed an almost complete loss of mechanically gated currents within our stimulus range (0–4,200 nm) (Fig. 5a,b). Next, N2a cells transfected with EGFP-tagged WT STOML3 were treated with or without MβCD. As previously reported^3^, overexpression of STOML3 robustly potentiated the mechanosensitive currents in N2a cells (Fig. 5c,d). However, this effect was substantially attenuated by depleting cholesterol with MβCD, indicating that STOML3 modulates Piezo1-mediated currents via the cholesterol-rich lipid raft.

We next determined whether STOML3 associating with cholesterol is important for its function in sensitization of Piezo1 channel activity. Expression of STOML3-P40S mutant deficient in binding cholesterol failed to potentiate Piezo1 currents in N2a cells, while depletion of cholesterol did not show any further effect (Fig. 5e,f). Thus, STOML3 modulates Piezo1 channel activity via association with cholesterol.

We next co-expressed Piezo2 channel with STOML3 in human embryonic kidney (HEK)293 cells. Similar to the results with Piezo1, MβCD treatment also attenuated the potentiation effect of STOML3 on Piezo2-mediated mechanosensitive currents, while expression of STOML3-P40S mutant failed to potentiate Piezo2 currents (Fig. 5g,h). Thus, STOML3 modulates both Piezo1 and Piezo2 channels activity via association with cholesterol. As reported in a previous study^41^, we also observed that the Piezo2 channel is much more difficult to express than the Piezo1 channel, possibly due to the cellular toxicity of heterologous overexpression of Piezo2. Since we observed that STOML3 exhibited a similar effect on Piezo1- and Piezo2-mediated mechanosensitive currents, we used Piezo1 for heterologous expression in the following experiments.

### STOML3 modulates membrane mechanics via binding cholesterol

**Figure 6:**
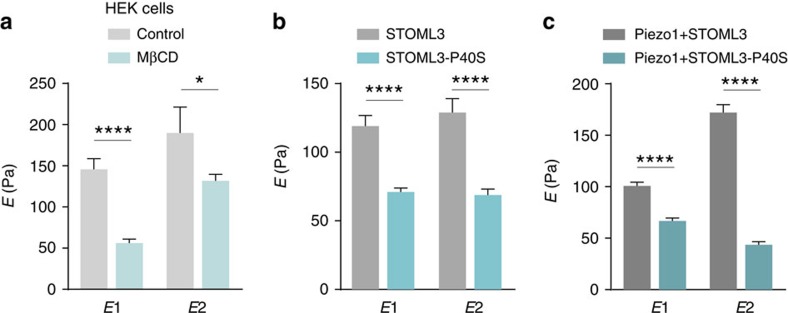
STOML3 modulates Piezo1 channel's surrounding membrane mechanics via association with cholesterol. (**a**) Quantitative comparison of elasticity modulus *E*_1_ and *E*_2_ values obtained by fitting procedure in HEK293 cells treated with or without MβCD (control: *n*=16; MβCD: *n*=17; two-way ANOVA with *post hoc* Bonferroni's test, *P*<0.0001). (**b**) Quantitative comparison of elasticity modulus *E*_1_ and *E*_2_ values obtained by fitting procedure in HEK293 cells expressing STOML3 or STOML3-P40 (STOML3: *n*=25; STOML3-P40: *n*=23; two-way ANOVA with *post hoc* Bonferroni's test, *P*<0.0001). (**c**) Quantitative comparison of elasticity modulus *E*_1_ and *E*_2_ values obtained by fitting procedure in HEK293 cells expressing STOML3 or STOML3-P40 in the presence of Piezo1 channel (Piezo1+STOML3: *n*=59; Piezo1+STOML3-P40: *n*=75; two-way ANOVA with *post hoc* Bonferroni's test, *P*<0.0001). *P<0.05, ****P<0.0001. Error bars indicate s.e.m.

Cholesterol depletion has been demonstrated to change membrane stiffness in rat sensory neurons^16^. However, it remains elusive whether the MβCD treatment displays a similar effect on HEK293 cell membrane. We therefore performed AFS measurements on HEK293 cells treated with or without MβCD. A significant reduction in cell stiffness after MβCD treatment was observed (Fig. 6a), suggesting that modifying membrane cholesterol level affects cell mechanics in HEK293 cells. We next ask whether the mechanical properties of membrane in which Piezo channels are embedded are controlled by STOML3 via associating with cholesterol. To address this question, HEK293 cells expressing STOML3 WT (STOML3-EGFP) or STOML3-P40S mutant (STOML3-P40S-EGFP) were subjected to AFS measurements. Analysis of force–indentation curves demonstrated that cells expressing STOML3-P40S mutant were significantly softer than cells with STOML3 WT (Fig. 6b). Interestingly, the reduction effect of STOML3-P40S mutant on the cell stiffness was comparable to the effect of cholesterol depletion, implying that the difference of cell stiffness between STOML3 WT and STOML3-P40S mutant-transfected HEK293 cells are mainly due to the ability of STOML3 associating with cholesterol. However, expressing STOML3 alone in HEK293 cells did not change the membrane stiffness (Supplementary Fig. 7). This may be due to the endogenous expression of other cholesterol-binding proteins, such as stomatin.

Piezo1 protein is reported to be extremely large with more than 30 predicted membrane spanning segments^42^. However, no alteration of membrane stiffness was observed by expression of Piezo1 alone (Supplementary Fig. 7). Next, we expressed Piezo1 channel together with STOML3 WT or STOML3-P40S mutant in HEK293 cells. Similar to the result obtained from STOML3-P40S mutant expression alone, the cell stiffness was significantly reduced in cells co-expressing Piezo1 and STOML3-P40S mutant when compared with cells co-expressing Piezo1 and STOML3 WT (Fig. 6c). These data suggest that STOML3 associates with cholesterol and modulates cell stiffness regardless of the presence of the Piezo1 channel. Thus, in a heterologous expression system, the interaction between STOML3 and cholesterol appears to be necessary for maintaining the membrane stiffness to enable efficient force transfer to Piezo channels.

### Cholesterol depletion attenuates tactile allodynia in mouse

**Figure 7:**
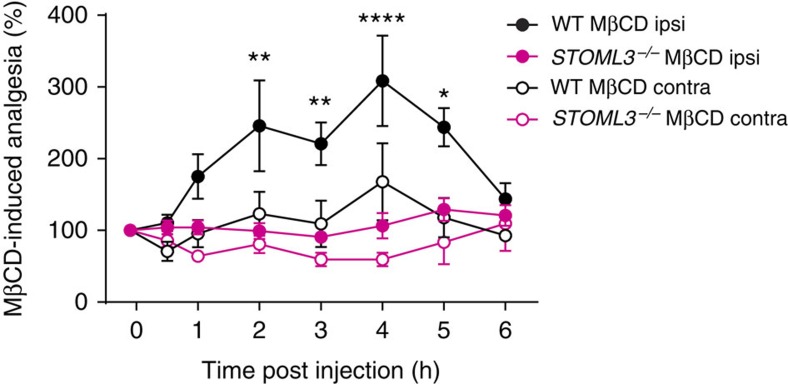
The *in vivo* effect of cholesterol depletion on tactile allodynia of *STOML3*^−/−^ mice. MβCD presented an analgesic effect on nerve injury-induced tactile allodynia in C57BL/6N, but not in *STOML3*^−/−^ mice (*n*=6–12 mice per group, MβCD effect: one-way ANOVA with *post hoc* Bonferroni's multiple comparison test, C57BL/6N: ipsilateral, *P*<0.05; contralateral, *P*>0.05; *STOML3*^−/−^: ipsilateral, *P*>0.05; contralateral, *P*>0.05; STOML3 influence: C57BL/6N versus *STOML3*^−/−^, two-way repeated measures ANOVA with *post hoc* Bonferroni's test, *P*<0.001). *P<0.05, **P<0.01, ****P<0.0001. Error bars indicate s.e.m.

To test the importance of cholesterol binding in mechanosensitivity *in vivo*, measurable tactile allodynia was induced by nerve injury. Control C57BL/6N mice and *STOML3*^−/−^ mice were subjected to chronic constriction injury (CCI) and developed a reduced paw withdrawal threshold to von Frey filament stimulation. A low dose of MβCD (2 mg kg^−1^) was injected to the hind paw on the injured side. Intriguingly, we observed that MβCD began to cause an attenuation of allodynia in control mice 2 h post injection and reached the peak effect 4 h post injection (Fig. 7; one-way ANOVA with a *post hoc* Bonferroni's multiple comparison test, *P*<0.05). Consistent with previous report about raft modulation on channel function^43^, our *in vitro* data showed that cholesterol depletion slightly but significantly depolarized the resting membrane potential in mechanoreceptors (Supplementary Table 4); thus the *in vivo* analgesic effect of MβCD we observed here is unlikely due to the reduction of mechanoreceptor excitability. However, we also observed a reduction of voltage-gated inward current density in nociceptors (Supplementary Fig. 8). To exclude the possibility that the attenuation of tactile allodynia was attributed to this, we have examined the effect of MβCD on acute pain behaviour. Administration of MβCD exhibited no alteration on the paw withdrawal threshold, indicating that the low dose of MβCD is not capable of affecting nocifensive behaviour under normal conditions (Supplementary Fig. 9). Remarkably, when we tested *STOML3*^−/−^ mice, the analgesic effect of MβCD on tactile allodynia was completely absent (Fig. 7; one-way ANOVA with a *post hoc* Bonferroni's multiple comparison test, *P*>0.05), strongly suggesting that lipid raft could regulate mechanosensitivity and this regulation requires the involvement of STOML3, or in another words, cholesterol association is required for the proper function of STOML3 in mechanosensitivity.

## Discussion

Our data reveal a novel mechanism by which STOML3 modulates sensory mechanostransduction: via associating with cholesterol-rich lipid raft, STOML3 controls the membrane mechanics, and thus facilitates the force transfer and tunes the sensitivity of the mechanically gated channels, including Piezo channels. Several lines of evidence support this conclusion. First, STOML3 was detected in a cholesterol-enriched lipid raft. Depletion of cholesterol and deficiency of STOML3 in sensory neurons similarly and interdependently affect membrane mechanics and attenuate mechanosensitivity. Second, we demonstrate that an intact STOML3 is essential for maintaining membrane mechanics to sensitize mechanically gated Piezo1 and Piezo2 channels in heterologous systems. Importantly, such a stiffened membrane can be softened by either depleting cholesterol or genetic disruption of cholesterol binding of STOML3. Third, mutation of the residue involved in cholesterol binding (STOML-P40S) failed to modulate the sensitivity of Piezo channels or restore the mechanosensitivity of *STOML3*^−/−^ sensory neurons. Finally, using a behavioural test, we show that cholesterol depletion could attenuate tactile allodynia and this effect involves STOML3. Factoring all these together, we suggest that the stiffened membrane by STOML3 binding cholesterol is essential for sensory mechanotransdution *in vitro* and the mechanical sensitivity *in vivo*.

Cholesterol-binding properties of MEC-2 are needed for both mechanosensitive channel activation *in vitro* and touch sensitivity in *C. elegans*^28^,^29^. Podocin, a MEC-2 homologue in mammals, has been suggested to be part of a mechanosensitive protein complex at the slit diaphragm of podocytes. Similar to MEC-2, podocin requires cholesterol to regulate stretch-activated channel TRPC6 activity^29^. Yet, it remains elusive how association of MEC-2 and podocin with cholesterol would contribute to mechanotransduction. STOML3, another mammalian homologue of MEC-2, has been shown to be essential for sensory mechanotransduction in mouse^2^. However, compared with other stomatin-like protein family members, for example, MEC-2 and podocin, even less is known about how STOML3 regulates mechanotransduction. Here, we first present data showing that STOML3 was detected in the cholesterol-rich lipid raft and that association with cholesterol is required for its proper function in regulating sensitivity of mechanically gated ion channels, including Piezo1 and Piezo2. Next, through AFS experiments, we discovered that the absence of STOML3 in sensory neurons selectively renders the plasma membrane softer than the WT, increases the viscosity and decreases the membrane tension. Previously, it has been shown that mutating the conserved proline of MEC-2 disrupts cholesterol binding^29^. Here we show that this proline residue is also essential for the association between STOML3 and cholesterol-enriched lipid raft. However, this may not necessarily suggest that the conserved proline residue P40 directly binds cholesterol. Kadurin *et al*.^36^ have suggested that the proline is essential for STOML3 hairpin-loop topology; mutating the proline residue to serine transfers STOML3 to a single-pass transmembrane form, and consequently renders STOML3 unable to interact with cholesterol. Compared with the single-pass transmembrane form, the hairpin-loop formed STOML3 and the cholesterol they gathered were mainly in the inner leaflet of the plasma membrane, which may have generated asymmetry in the shape and force distribution of the plasma membrane^9^. This is consistent with our AFS measurements in HEK293 cells that the reduction effect of STOML3-P40S mutant on the cell stiffness was comparable to the effect of cholesterol depletion. Anishkin and Kung^9^ have suggested in a recent review that cholesterol, together with sphingomyelin and specialized proteins, forms a stiffened platform that can redirect, rescale and confine force. Our results suggest that cholesterol recruited by STOML3 is necessary for maintaining the stiffened membrane surrounding the mechanically gated ion channel and facilitating tension transmission in mouse sensory neurons. Dissolving this stiffened membrane, for example, by depletion of cholesterol, may require stretching the entire plasma membrane to activate mechanosensitive channels. Cholesterol recruitment to the associated ion channels is an intrinsic function of stomatin-like proteins, such as MEC-2 and podocin. This implies that MEC-2 and podocin may share a similar mechanism as STOML3, creating a specialized local membrane environment with distinct physical properties that enables the function of associated mechanically gated ion channels. Accordingly, the ion channel needs to be targeted to the stiffened membrane through the interaction with specific stomatin-like proteins, for example, MEC-4 through MEC-2, TRPC6 through podocin and Piezo channels through STOML3. This is in line with the reported observation that neither podocin nor MEC-2 could regulate the mechanosensitivity of Piezo channels^3^.

Mechanical gating controlled by a membrane force profile has been well established for the prokaryotic stretch-activated channels MscL and MscS^44^,^45^. Recently, neuronal mechanics has also been shown to contribute to touch sensation in *C. elegans*^10^,^17^, however, it is not clear whether the activity of eukaryotic mechanosensitive channels can be directly modulated by membrane mechanics. Interestingly, we observed that alteration of membrane mechanics either by depletion of cholesterol or deficiency in STOML3 could regulate the sensitivity of both SA and RA channels in mouse sensory neurons. Thus, the membrane mechanics not only modulates stretch-activated channel (SA) sensitivity, but also affects the activity of the tether gated ion channel (RA). By binding STOML3, the ordered cholesterol-rich lipid rafts could associate with mechanosensitive ion-channel complexes, for instance, the stretch-activated SA channel complex, thus modulating its local membrane environment and facilitating force transfer. Disrupting the associated lipid rafts does not disassemble the complex but may require stretching the entire plasma membrane to activate the mechanosensitive SA channel^9^,^28^,^43^. By recruiting cholesterol to the associated ion channels, STOML3 may alter the local membrane mechanics, influence the free energy of channels and set the availability of mechanosensitive channels, such as the tether-activated RA channel, thus determining its current amplitude when gated by force. This is similar to the effect of MEC-2 on sensory mechanotransduction channels MEC-4/MEC-10 in *C. elegans*, where the function is altered by increasing the number of channels in an active state rather than by markedly affecting either single-channel properties or surface expression^28^. It is also possible that STOML3, by binding cholesterol and forming higher-order scaffolds, may redistribute and concentrate mechanically gated ion channels accessible for force transmitting protein in lipid rafts.

In our behaviour experiment, we observed that cholesterol depletion attenuated the tactile allodynia in mouse. After nerve injury, the development of tactile allodynia has been suggested mostly due to the crosstalk between low-threshold mechanoreceptors and the nociceptive circuits in the superficial dorsal horn^46^,^47^. However, evidence for the sensitization of mechanotransduction in nociceptors is also emerging^47^. The majority of low-threshold mechanoreceptors carry Piezo2-dependent RA currents, while mechano-nociceptors carry a mixed repertoire of RA, IA and SA currents. As our electrophysiological results showed, depletion of cholesterol could regulate the sensitivity of both SA and RA channels in mouse sensory neurons. So the MβCD-mediated attenuation of mechanical sensitivity might be due to regulation of the mechanosensitivity of either the low-threshold mechanoreceptors or the mechano-nociceptors. This specifies an advantage of cholesterol depletion as a potential chronic pain treatment, which is independent of the mechanism of tactile allodynia development.

Altogether, our data provide a novel mechanism by which STOML3, via binding cholesterol, gathers mechanosensitive channels in lipid rafts, facilitates force transfer and regulates the activity of different mechanosensitive channels regardless of how they are gated by force. Thus, targeting STOML3–cholesterol association might offer a unique peripheral means of chronic pain control.

## Methods

### Animals

For DRG neurons transfection, experiments were carried out in neonatal male *STOML3*^−/−^ mice (aged 1–3 weeks). For all the other experiments, adult C57/Bl6N and *STOML3*^−/−^ male mice (aged 8–13 weeks) were used. Mice were housed under a 12:12-h light/dark cycle. All animal experiments were done according to the German Animal Protection Law. The Ethics Committee from Regierungspresidium Tuebingen, Germany, approved this study.

### Molecular biology

RNA was isolated from adult C57/Bl6 mice using the RNA extraction kit (Qiagen). The resulting 2 μg of poly(A)-RNA was reverse transcribed using SuperScript II reverse transcriptase (Invitrogen), followed by PCR. For cloning of mouse *STOML3*, the following primer pairs were used (Gen-Bank accession number NM_153156): 5′-AGGCACCTCAAGAATGAGATGG-3′ (forward) and 5′-CAGGCTGTTACGTGGAAGACC-3′ (reverse). The full-length open-reading frames of mouse *STOML3* were then subcloned into pcDNA3.1(+) expression vector (Invitrogen), Myc (E Q K L I S E E D L) sequences were tagged at the C terminus of *STOML3*. Mouse *STOML3* complementary DNA (cDNA) was also subcloned into pEGFP-N1 expression vector. Proline at the 40th residue was mutated to serine in *STOML3* (STOML3-P40S) by recombinant PCR.

### DRG neurons culture and transfection

DRG neurons from adult male C57/Bl6N and *STOML3*^−/−^ mice were prepared as previously described^4^. Briefly, DRGs were dissected and collected in a 1.0-ml tube of PBS on ice. Ganglia were cleaned, enzymatically treated and mechanically dispersed. Before plating on poly-L-lysine-laminin-coated coverslips, cells were transfected using the Nucleofector system (Lonza AG). In brief, neurons were suspended in 20 μl of Mouse Neuron Nucleofector solution from the SCN nucleofector kit (Lonza AG) and a total 4–5 μg of plasmid DNA at room temperature. The mixture was transferred to a cuvette and electroporated with the preinstalled program SCN Basic Neuro program 6. After electroporation, the cell suspension was transferred to 500 μl of RPMI 1640 medium (Gibco) for 10 min at 37 °C. This suspension, supplemented with 10% horse serum, was used to plate the cells onto glass coverslips for recording. The RPMI medium supplemented with 100 ng ml^−1^ nerve growth factor and 50 ng ml^−1^ BDNF was replaced with the standard DRG medium 3–4 h later. Electrophysiology experiments began 12 h after plating.

### Cell line culture and transient transfection

CHO cells (Leibniz Institute DSMZ-German Collection of Microorganisms and Cell Cultures, Germany), HEK293 cells (Leibniz Institute DSMZ-German Collection of Microorganisms and Cell Cultures) and mouse neuroblastoma N2a cells (a kind gift from Prof. Burkhard Schlosshauer in NMI, Germany) were cultured at 37 °C, 5% CO_2_ in DMEM nutrient medium (GIBCO) supplemented with 10% fetal bovine serum and 1% penicillin–streptomycin. Cells were transfected with cDNA using lipofectamine 2000 transfection reagent (Invitrogen) according to the manufacturer's recommendations. CHO cells used for isolating lipid rafts were cultured in 100-mm dishes, and transfected using 10 μg Myc-STOML3 or Myc-STOML3-P40S. N2a cells used for electrophysiological recording were plated at a roughly 50% confluence onto 12-mm round glass coverslips, and transfected with GFP (EGFP)-tagged STOML3 or STOML3-P40S. Piezo2-EGFP was co-transfected with Myc-STOML3, Myc-STOML3-P40S or empty pcDNA3.1(+) vector at a 1:5 ratio in HEK293 cells. Whole-cell patch-clamp recordings were performed 18–48 h after transfection. For stiffness measurement, Piezo1-IRES-GFP was co-transfected with Myc-STOML3, Myc-STOML3-P40S or empty pcDNA3.1(+) vector at a 1:8 ratio in HEK293 cells and were used 48 h later.

### Electrophysiology

Whole-cell patch-clamp recordings from isolated DRG neurons were made as previously described^4^,^5^,^6^. Recordings were made from DRG neurons using fire-polished glass electrodes with a resistance of 3–7 MΩ. Extracellular solution contained (mM): NaCl 140, MgCl_2_ 1, CaCl_2_ 2, KCl 4, glucose 4 and HEPES 10 (pH 7.4), and electrodes were filled with a solution containing (mM): KCl 130, NaCl 10, MgCl_2_ 1, EGTA 1 and HEPES 10 (pH 7.3). Cells were perfused with drug-containing solutions by moving an array of outlets in front of the patched cells (WAS02; Ditel, Prague). Observations were made with the Observer A1 inverted microscope (Zeiss, Germany) equipped with a charge-coupled device camera and the imaging software AxioVision. Membrane current and voltage were amplified and acquired using EPC-10 amplifier sampled at 40 kHz; acquired traces were analysed using Patchmaster and Fitmaster software (HEKA). Pipette and membrane capacitance were compensated using the auto function of Pulse. For most of experiments, to minimize the voltage error, 70% of the series resistance was compensated, the membrane voltage was held at −60 mV with the voltage-clamp circuit. After establishing whole-cell configuration, voltage-gated currents were measured using a standard series of voltage commands. Briefly, the neurons were prepulsed to −120 mV for 150 ms and depolarized from −65 to +55 mV in increments of 5 mV (40 ms test pulse duration). Next, the amplifier was switched to current-clamp mode and current injection was used to evoke action potentials. If the membrane capacitance and resistance changed more than 20% after the mechanical stimulus, the cell was regarded as membrane damaged and the data discarded. Mechanical stimuli were applied using a heat-polished glass pipette (tip diameter 3–5 μm), driven by a MM3A Micromanipulator system (Kleindiek) and positioned at an angle of 45 degrees to the surface of the dish. The probe was positioned near the neurite, moved forward in steps of 200–600 nm for 500 ms and then withdrawn. For analysis of the kinetic properties of mechanically activated current, traces were fit with single exponential functions using the Fitmaster software (HEKA). Data are presented as mean±s.e.m.

### Force spectroscopy measurements

Force spectroscopy measurements were performed using a NanoWizard AFM (JPK Instruments, Berlin, Germany) equipped with a fluid chamber (Biocell; JPK) for live cell analysis and an inverted optical microscope (Axiovert 200; Zeiss) for sample observation. DRG neurons or HEK cells were inserted into the fluid chamber immersed in culture medium and measurements were carried out at room temperature. The status of cells was constantly monitored by optical microscope. Indenters for probing cell elasticity were prepared by mounting silica microspheres of 4.5 μm nominal diameter to silicon AFM tipless cantilevers using ultraviolet-sensitive glue. The tipless V-shaped silicon nitride cantilevers have nominal spring constants of 0.32 or 0.08 N m^−1^ (NanoWorld, Innovative Technologies). Silica beads were picked under microscopy control. Before measurements, the spring constant of the cantilevers was calibrated using the thermal noise method^48^. Nano-indentation measurements were performed to evaluate cell elasticity. Using an optical microscope, the bead-mounted cantilever was brought over the soma of single DRG and pressed down to indent the cell. The motion of the *z*-piezo and the force were recorded. On each cell eight-about ten force–displacement (*F*–*D*) curves were acquired with a force load of 0.5 nN and at a rate of 5 μm s^−1^ in closed-loop feedback mode. Analogous measurements were carried out on transfected HEK293 cells. In this case, cells were selected using fluorescence microscope: only those cells showing fluorescence signal were used for indentation measurements.

For evaluation of DRG cell membrane viscosity, the force needed to pull a plasma membrane tether (that is, tether force) at a constant speed is measured and then the dependence of tether force on speed is analysed. In these measurements, the bead-mounted cantilever was pressed against the cell soma with a contact force of 500 pN for contact time of 10–30 s to allow the cell establishing adhesion points with the silica bead. Afterwards, the cantilever was retracted with different pulling speed (2, 5, 8, 12 and 18 μm s^−1^). After each force measurement, the cell was allowed to recover for a time period comparable to the contact time, before performing further pulling measurements. The resulting *F*–*D* retraction curves present a long plateau with step-like features whose height provides a direct measure of tether force.

### Evaluation of cell elasticity

Cell elastic properties can be assessed by determining the Young's modulus. The Hertz model describes the relation between the applied force (*F*), indentation (*δ*) and Young's modulus (*E*). To evaluate *E* from the force–indentation curves, a Sneddon's modification of the Hertz model for the elastic indentation of a flat, soft sample by a stiff sphere can be used^49^.


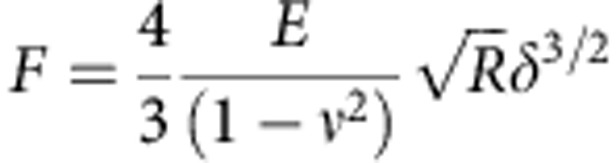


However, a cell comprises of different structures starting from the external plasma membrane, cortical actin, cytoskeleton, cytoplasmic organelles and nucleus, which make it a complicated mechanical system. The deformations of different cell structures could determine different regimes, which will appear convoluted into a single force–indentation curve. The total indentation in this case would comprise of two (or even more) contributions: the first from the compression of the superficial layer of the cell, while the other corresponding to the rest of cell structures (that is, cytoskeleton, nuclei and so on). To take into account the presence of such different contributions, we used a variation of this model. We consider the cell as a set of elastic components, where first it has indented the external layer and then the second internal one (Supplementary Fig. 2). If we assume that each single part deforms elastically, the fitting curve can be written as follows:


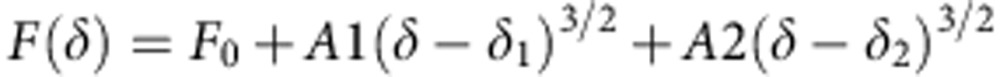



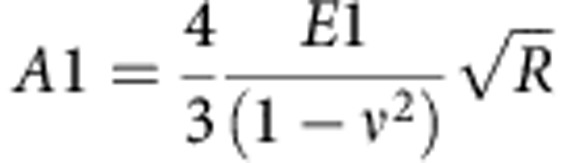



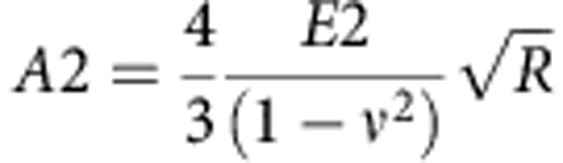


where *F*_0_ is the force at zero deformation, *R* is the radius of the spherical indenter and *ν* is the sample's Poisson ratio (set to 0.5 for the cell)^50^. *E*_1_ and *E*_2_ are the Young's modulus as obtained for the first and second elastic component of the curve, respectively, while *δ* is the indentation. *δ*_1_ and *δ*_2_ represent the first and the second contact point, respectively. The first contact point, defined as the point where the cantilever deflection starts to rise and the second point where the curve changes regime. Hence, the second contact point, as revealed by the fitting procedure, can be used to identify the regime variation in the curve (Supplementary Fig. 2).

Data fitting was performed on the approach part of the recorded *F*–*D* curves. JPK DP software was used to convert the curve into the force*–*indentation curve by subtracting the cantilever bending from the signal height to calculate indentation. The total number of curves for WT and *STOML3*^−/−^ sensory neuron was then fitted by a MatLab routine. Resulting *E*_1_ and *E*_2_ values were plotted as a bar graph and statistically analysed.

In those cases in which significant changes in regime were not observed and the fit provided a second contact point outside the experimental data range, only the *E*_1_ value is provided for the whole cell (examples are shown in Supplementary Fig. 3).

According to this fitting procedure, we could identify a second contact point at 360±10 nm for WT and 330±10 nm for *STOML*3^−/−^ (± indicates s.e.m.). In total, 31% of the curves for WT DRG and 41% for *STOML*3^−/−^ displayed two different regimes in the fitting range of the force–indentation curve (−1 and +1 μm). This suggests that in some cases, the superficial layer of the cell has significantly different elastic properties from the rest of the cell, while in other cases the cell behaves as a homogenous elastic material and the fit can be performed with a classical Hertz model for a homogeneous material.

In the case of HEK293 cells transfected with empty vector, the second contact point was found at 420±20 nm (± indicates s.e.m.), consistent with all the other transfected groups analysed.

### Evaluation of DRG neuron membrane viscosity and tension

For evaluating cell membrane viscosity, the mean tether force per cell was obtained and then the average on 20 cells was plotted as a function of the pulling velocity. The effective viscosity *η*_eff_ and threshold pulling force *F*_0_ were then obtained by a linear fit of the force–velocity curve^51^


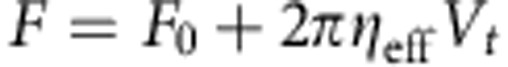


The *F*_0_ threshold force for extracting a tether is given by


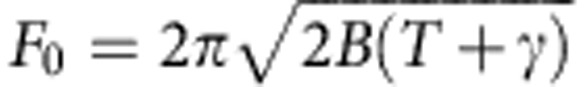


where *B* is the membrane-bending stiffness, *T* is the in-plane membrane tension and *γ* is membrane–cytoskeleton adhesion energy. The sum (*T*+*γ*) provides the apparent surface tension (*T*_app_). Hence using the formula: *T*_app_=*F*_0_^2^/(8 *πB*), we evaluated the apparent surface tension considering a *B*-value of 2.7 × 10^−19^ Nm (ref. 51). Percentage of error has been evaluated according to propagation of error.

### Cholesterol depletion

Cholesterol depletion was performed as described previously^52^. Briefly, raft-like microdomains were chemically disrupted by depleting cholesterol with MβCD (Sigma). In all cases, cells were washed twice with PBS and then incubated with 5 mM MβCD. DRG neurons and N2a cells were incubated at 37 °C for 1 h in 5 mM MβCD. HEK293 cells were incubated at 37 °C for 0.5 h in 5 mM MβCD. For visualizing cholesterol, neurons were incubated with Filipin (50 μg ml^−1^ in PBS; Sigma), a cholesterol-binding fluorescent agent, for 2 h after fixation with 4% paraformaldehyde in PBS for 10 min at room temperature.

### Isolation of lipid rafts and immunoblotting

Lipid rafts from transfected CHO cells were obtained as described previously^52^,^53^. Briefly, transiently transfected cells were homogenized in 1% Triton X-100 containing lysis buffer (20 mM HEPES, 5 mM EDTA, 150 mM NaCl, pH 7.4) with Complete protease inhibitors. The solution was then lysated by ultrasonic and 500 μl of lysate would be mixed with 500 μl of 80% sucrose solution (final concentration, 40%) and applied in the bottom of a centrifugation tube. A discontinuous sucrose gradient was prepared by layering 2 ml 35% sucrose and 1 ml 5% sucrose. Gradients are centrifuged at 339,300*g* for 22 h in a Thermo TH-660 rotor at 4 °C in a WX80 Ultracentrifuge. After centrifugation, eight fractions (500 μl) from the top were collected, and equal volumes of each fraction were analysed by western blotting. The endogenous raft-specific protein flotillin-2 was used to check the effectiveness of this method, which is supposed to be expressed in buoyant, low-density fractions.

### Immunoblotting

Samples were separated by 10% SDS–polyacrylamide gel electrophoresis gel and transferred to a polyvinylidene difluoride membrane (Roche). The membrane was blocked in 5% bovine serum albumin in TBST (10 mM Tris, 150 mM NaCl and 0.1% Tween 20, pH 8.0) and then incubated with primary antibody: anti-c-Myc (1:3,000; Sigma; catalogue number: C3956), anti-Flotillin-2 (1:2,000; Santa Cruz; catalogue number: sc-25507). Membranes were subsequently incubated with horseradish peroxidase-conjugated anti-rabbit IgG (1:5,000; Santa Cruz; catalogue number: sc-2004) and developed with an enhanced chemiluminescence kit (Roche). Quantification analysis was performed with ImageJ software (NIH, Bethesda, MD; USA).

### Induction of neuropathic pain and nociceptive tests

CCI was induced as described^2^,^54^. In brief, in deeply anaesthetized mice (isoflurane) four loose silk ligatures (4/0) were placed (with about 0.5 mm spacing) around the sciatic nerve at the level of the left mid-thigh. Ligatures were tied until they elicited a brief twitch in the respective hindlimb. Mechanical allodynia was measured using von Frey filament (North Coast Medical Inc., CA, USA). Intraplantar injection of MβCD (Sigma, 2 mg kg^−1^) dissolved in sterile saline was performed 2 days after CCI. The withdrawal threshold was measured for each mouse before and at various times after injection. All experiments were performed blind to both the genotype of the mice and the injected drug.

### Data and statistical analysis

Fitmaster, Origin 8 and Graphpad Prism 6 software suites were used to perform linear and nonlinear fitting of data. The time course of current inactivation *τ* was fitted to the first-order exponential. Current–stimulus curves were fitted using the following Boltzman function: *I*(*x*)=*I*_max_[1+exp(*x*_1/2_*−x*)/*s*]^−1^, where *I* is the peak amplitude at holding potential −60 mV, *x* is the displacement (in nanometre) of the mechano-probe, *I*_max_ is the maximum current, *x*_1/2_ is the displacement value producing 50% of *I*_max_ and *s* is the slope sensitivity indicating the change in channel active-state probability. All statistical comparisons were two sided and were performed with Prism. For all *in vitro* experiment, recordings were pooled from at least three mice. The sample size was justified by significance testing, taking into account available number of neurons per *in vitro* experiment or mice. Unpaired two-tailed *t*-tests and two-way ANOVA were used for two-group comparison. One-way repeated measures ANOVA with *post hoc* Dunnett's multiple comparison test was used to judge MβCD effect over time. Two-way repeated measures ANOVA was performed to compare the MβCD effect on C57BL/6N and *STOML3*^−/−^ mice over time. *P*<0.05 was considered to be statistically significant. All means are expressed as mean±s.e.m. All replicates were biological, and all experiments were performed blind to the genotype of the mice.

## Additional information

**How to cite this article:** Qi, Y. *et al*. Membrane stiffening by STOML3 facilitates mechanosensation in sensory neurons. *Nat. Commun*. 6:8512 doi: 10.1038/ncomms9512 (2015).

## Supplementary Material

Supplementary Information Supplementary Figures 1-9, Supplementary Tables 1-4
