# Supplementary material for: Membrane stiffening by STOML3 facilitates mechanosensation in sensory neurons
Source: Nat Commun. 2015 Oct 7;6:8512. doi: 10.1038/ncomms9512 (PMC4633829; doi:10.1038/ncomms9512)
Supplement: Supplementary Information — Supplementary Figures 1-9, Supplementary Tables 1-4 [file ncomms9512-s1.pdf]

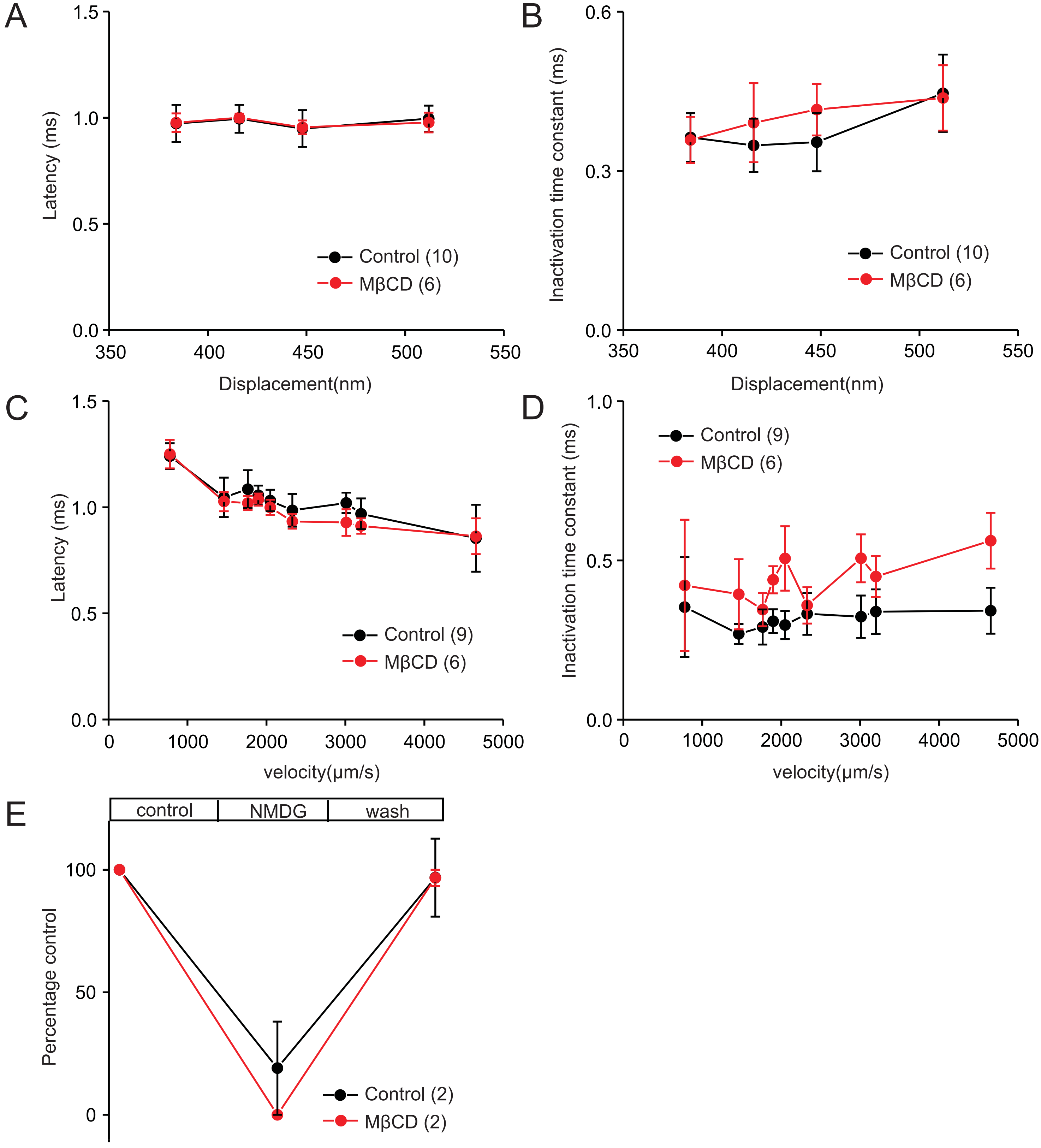

Supplementary Figure 1, Related to Figure 2

MβCD treatment did not alter the kinetic properties or the the ion selectivity of RA-mechanosensitive currents. The latency (A) and inactivation time constant (B) of mechanically activated RA currents plotted against the mechanical stimulus intensities for control and MβCD treated neurons. No significant difference was observed in the latency and inactivation time constant between control and MβCD-treated cells at each stimulus strength (Two-way repeated ANOVA followed by Bonferroni *post hoc* test, $p>0.05$). The latency (C) and inactivation time constant (D) of RA-mechanosensitive currents plotted against the stimulus velocities for control and MβCD-treated groups. No significant difference was observed in the latency and inactivation time constant between control and MβCD-treated cells at each velocity (Two-way repeated ANOVA followed by Bonferroni *post hoc* test, $p>0.05$). (E) MβCD treatment did not influence the ion selectivity of RA currents. The RA currents were largely abolished by the replacement of sodium ion in the extracellular buffer with the non-permeant cation $NMDG^+$ in both control and MβCD groups.The number of cells is noted. Error bar indicates s.e.m.

Supplementary Figure 1

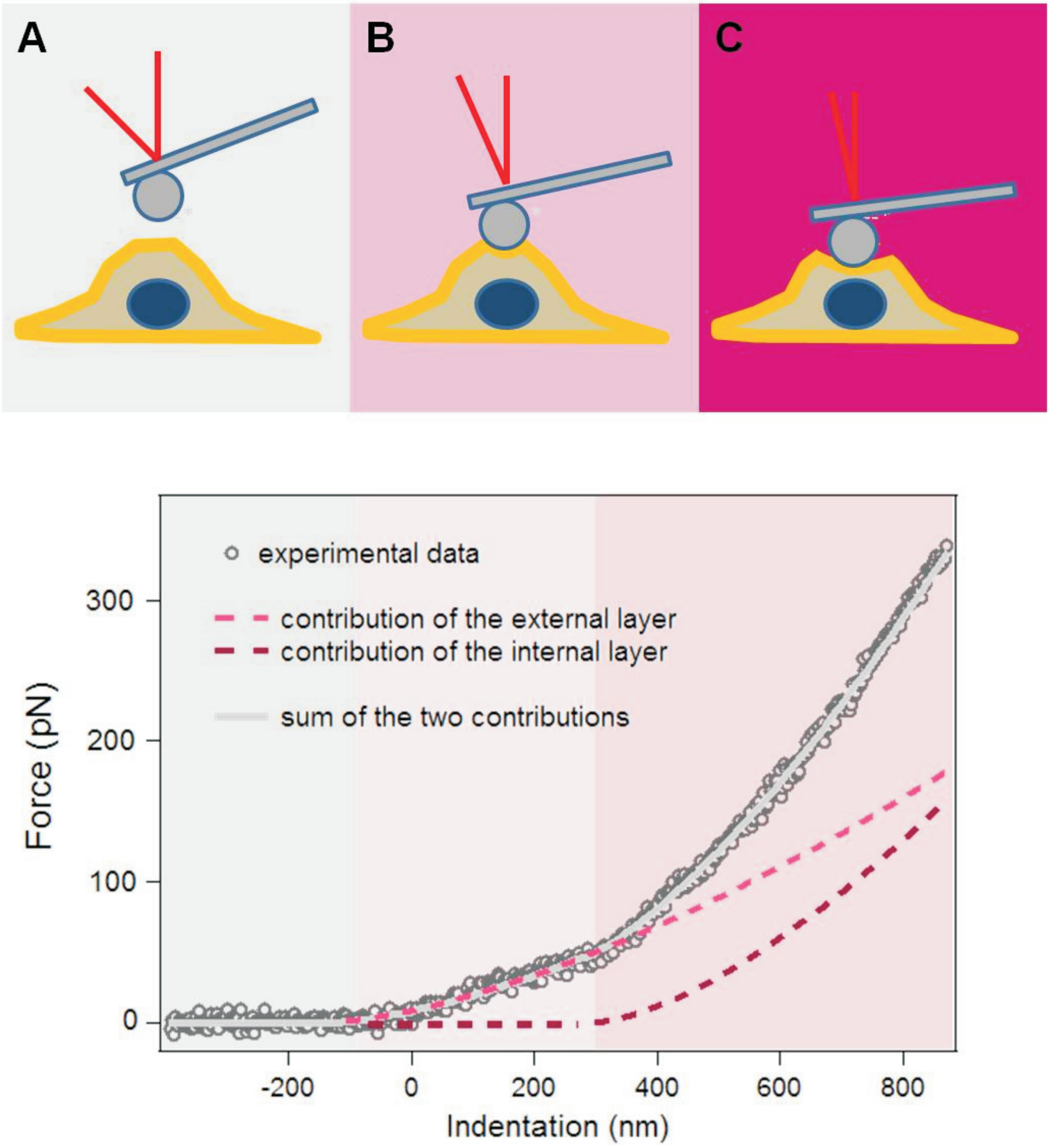

Supplementary Figure 2, Related to Figure 3

Sketch illustrating the indentation steps of the bead on a cell. In the upper panel it is shown the movement of the cantilever downward the sample and the cell indented by the bead. (A) before first contact point: no indentation forces are observed; (B) between first and second contact points: indentation forces are generated by the only contribution of the external layer; (C) after second contact point both external and internal layer contributes, each following the Hertzian law. In such a case the measured force indentation curve is the sum of these two contributions. In the lower panel the regimes for the different contributions and the resulting sum into a force-indentation curve are shown.

Supplementary Figure 2

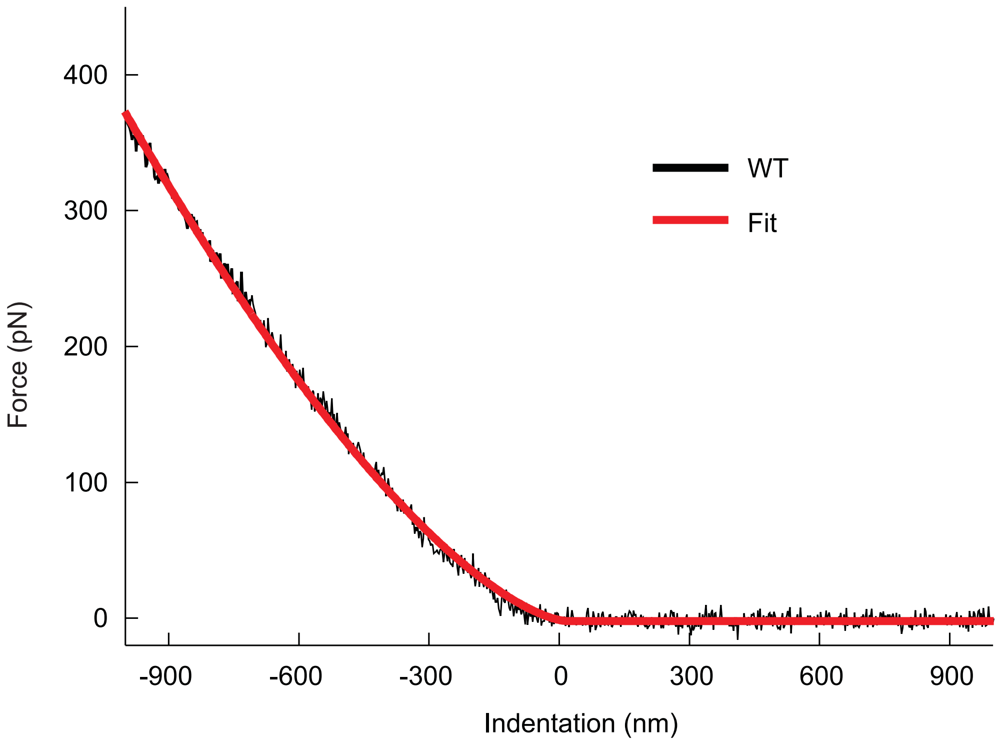

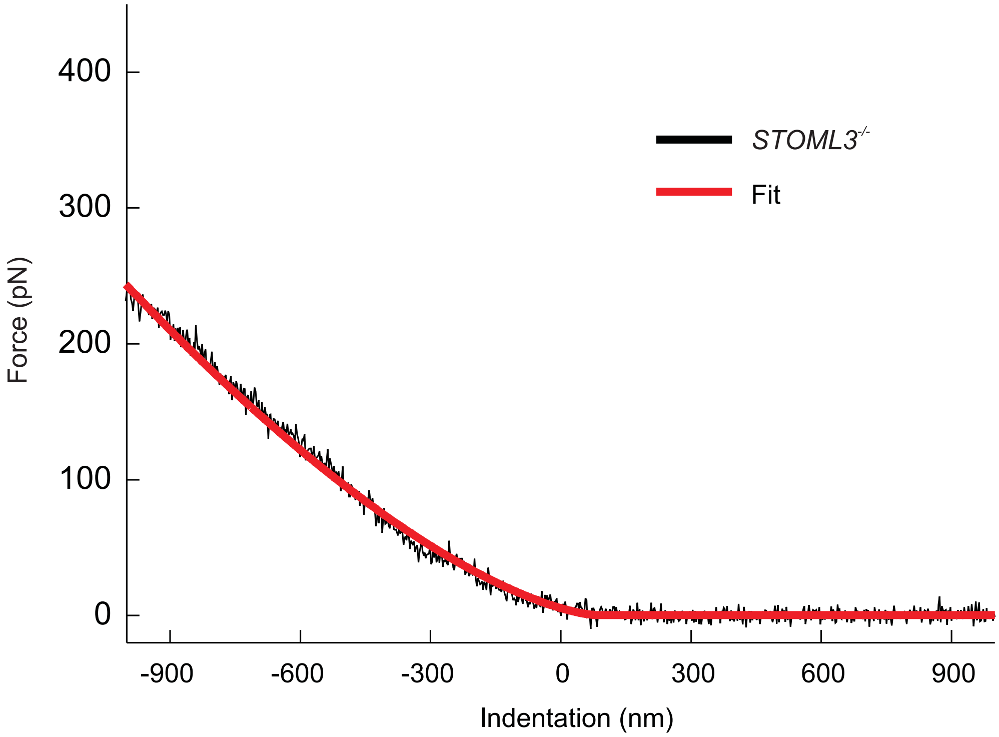

Supplementary Figure 3, Related to Figure 3
Examples of force-indentation data and fitting curve both for WT and *STOML3*$^{-/-}$ sensory neurons as fitted by Hertz model for a single homogenous regime

Supplementary Figure 3

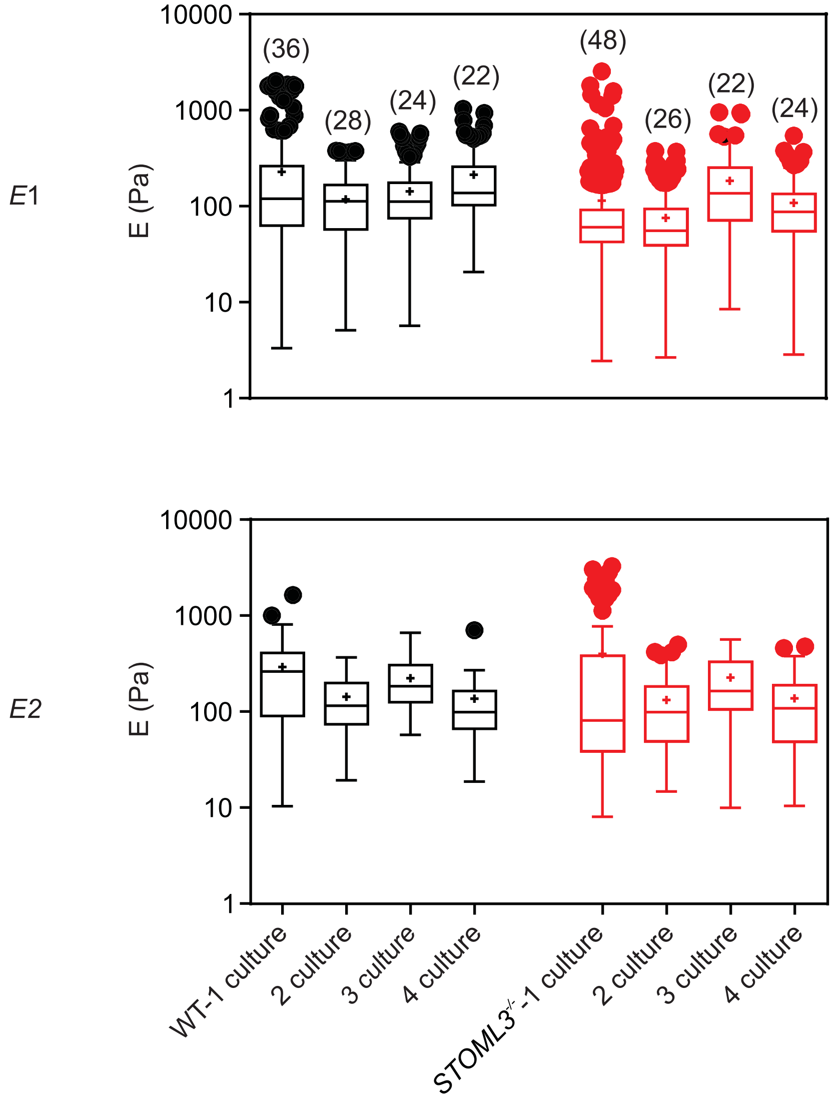

Supplementary Figure 4. Related to Figure 3

Distribution of elasticity modulus *E* values on WT and *STOML3*$^{-/-}$ sensory neurons. Elasticity modulus *E*1 and *E*2 obtained from four different cultures: values inside the box represent the first (25%) and third quartile (75%), the line within the box represents the median value, the (-) indicate the maximum and minimum observations, while outliers are indicated by (•), the mean value is indicated in the plot as (+). Y scale is reported as Log for a better visualization and comparison of the data. The number of cells analyzed is indicated in parentheses.

Supplementary Figure 4

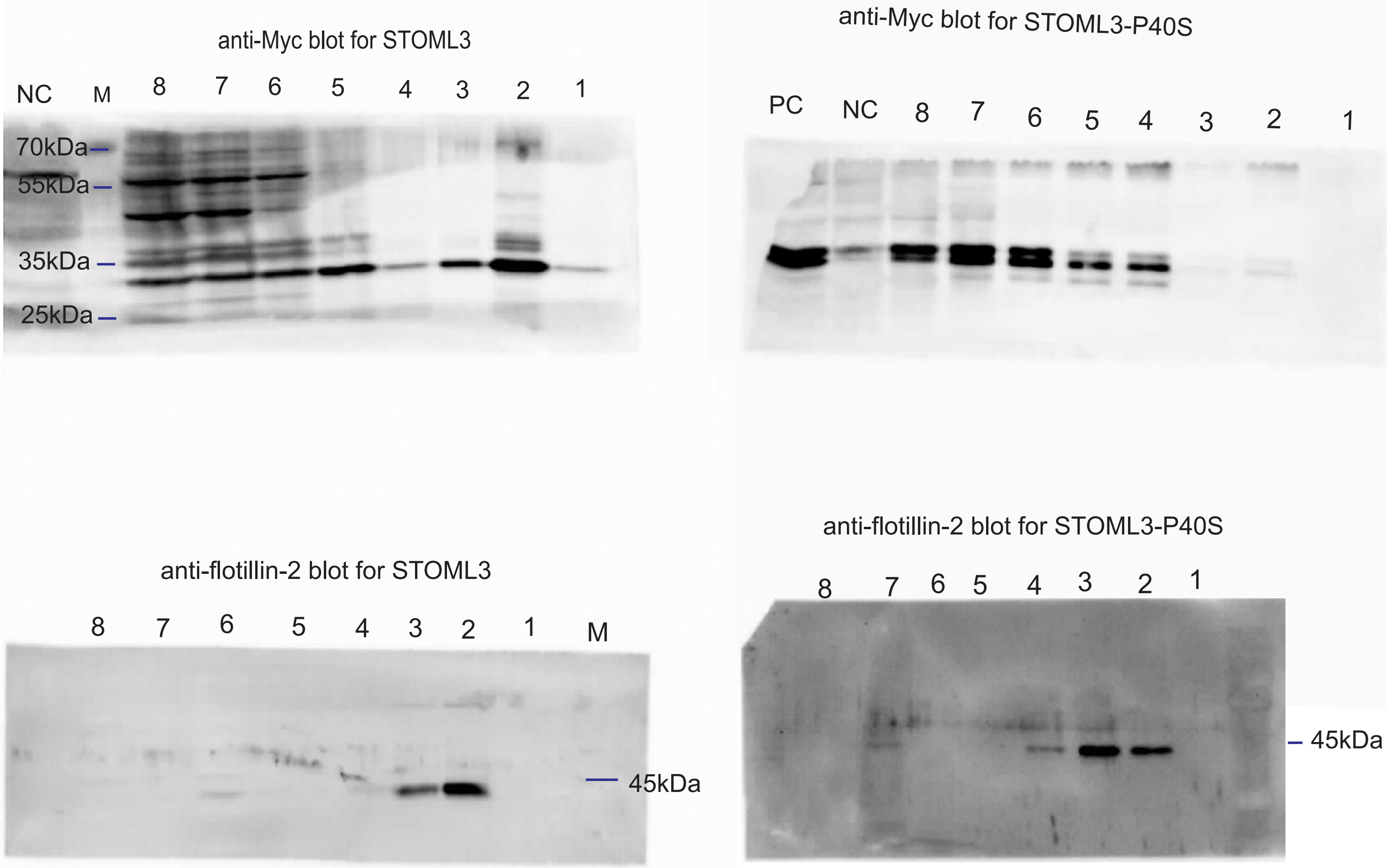

Supplementary Figure 5 , Original blots for the ones presented in Figure 4B

Supplementary Figure 5

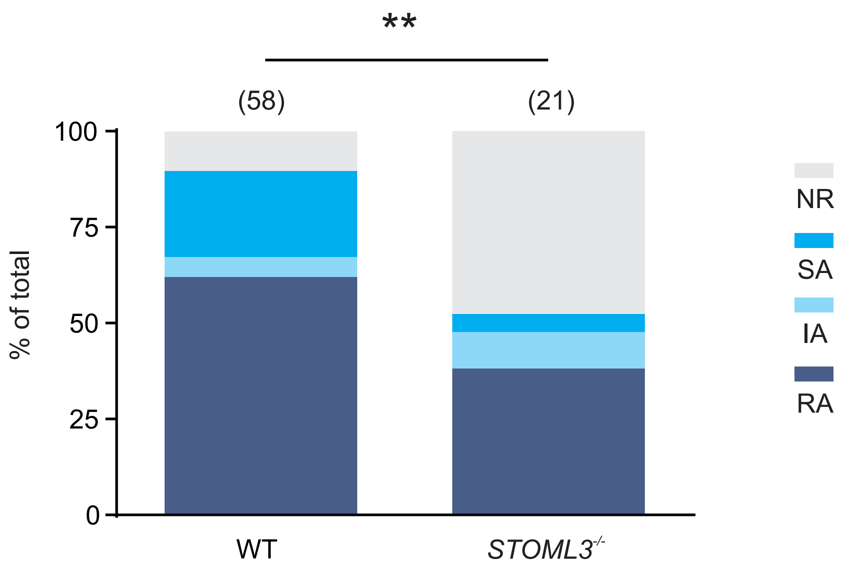

Supplementary Figure 6, Related to Figure 4

Absence of STOML3 reduces mechanosensitivity in sensory neurons. Stacked histograms showing the proportions of different mechano-gated currents observed in WT and *STOML3*$^{-/-}$ neurons. Note the marked loss of mechanosensitive currents in *STOML3*$^{-/-}$ neurons.(χ^2 test, P<0.01). The number of neurons recorded is indicated in parentheses in each panel. NR, non-responsive to given displacement 512nm.** *P*<0.01.

Supplementary Figure 6

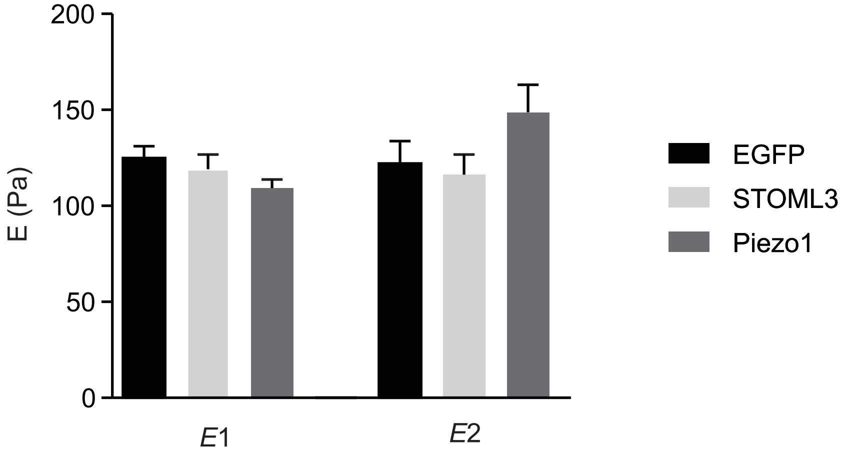

Supplementary Figure 7, Related to Figure 6

STOML3 or Piezo1 did not alter cell stiffness in heterologous expression system. Quantitative comparison of elasticity modulus *E*1 and *E*2 values obtained by fitting procedure in HEK293 cells expressing EGFP, STOML3 (STOML3-EGFP) or Piezo1 (Piezo1-IRES-EGFP). Neither STOML3 nor Piezo1 expression HEK293 cells altered cell stiffness when compared with cells expressing EGFP (EGFP: n=25; STOML3: n=25; Piezo1, n=59 ; two-way ANOVA with *post-hoc* Tukey's test, EGFP vs. STOML3, $P>0.05$; EGFP vs.Piezo1, $P>0.05$; STOML3 vs.Piezo1, $P>0.05$). Error bars indicate s.e.m.

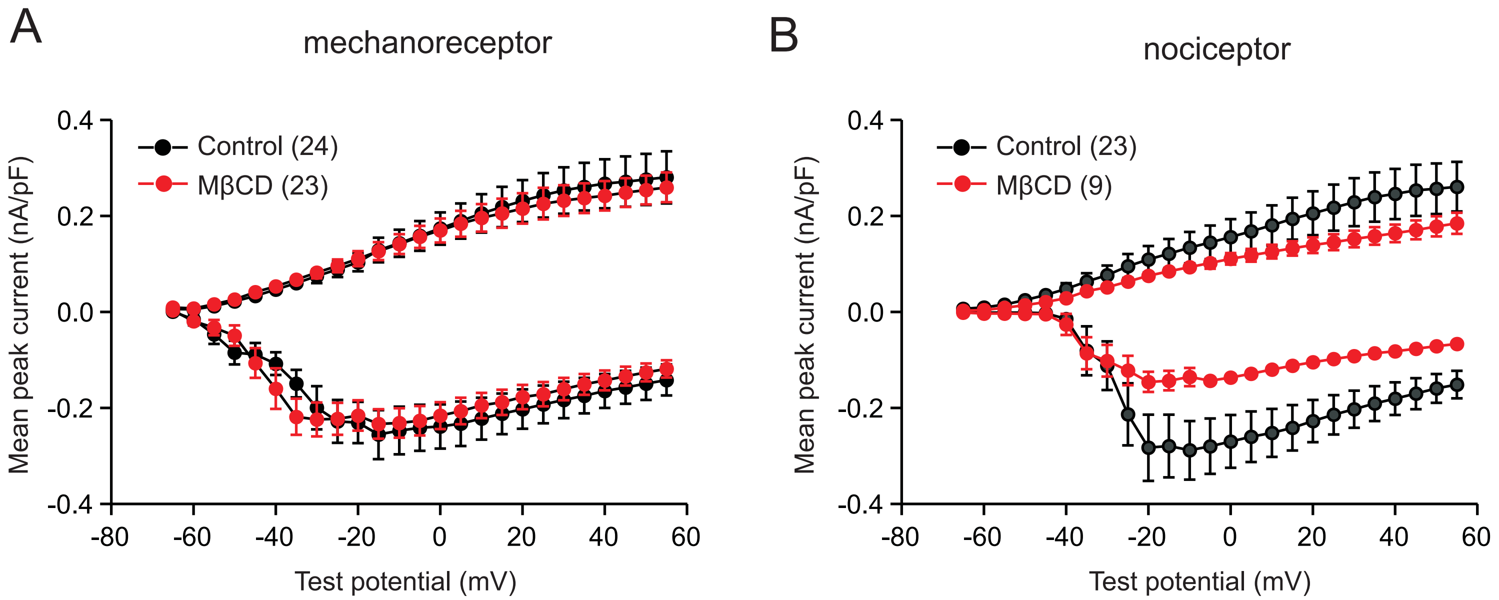

Supplementary Figure 8, Related to Figure 7

Effects of MβCD treatment on voltage gated currents in cultured sensory neurons. Mean whole-cell inward and outward currents measured at different test potentials for control and MβCD treated neurons. Neurons are grouped into mechanoreceptor (**a**) (1/2 AP duration < 1ms) and nociceptor (**b**) (1/2 AP duration >=1ms). Neurons were first prepulsed to -120mV and then depolarized from -65mV to 55mV in 5-mV increment. No significant change was seen in the peak amplitude of voltage gated inward and outward current between control and MβCD-treated cells at each test potential for mechanoreceptor or nociceptor (Two-way repeated ANOVA followed by Bonferroni *post hoc* test, p>0.05). The number of cells is noted. Error bar indicates s.e.m.

Supplementary Figure 8

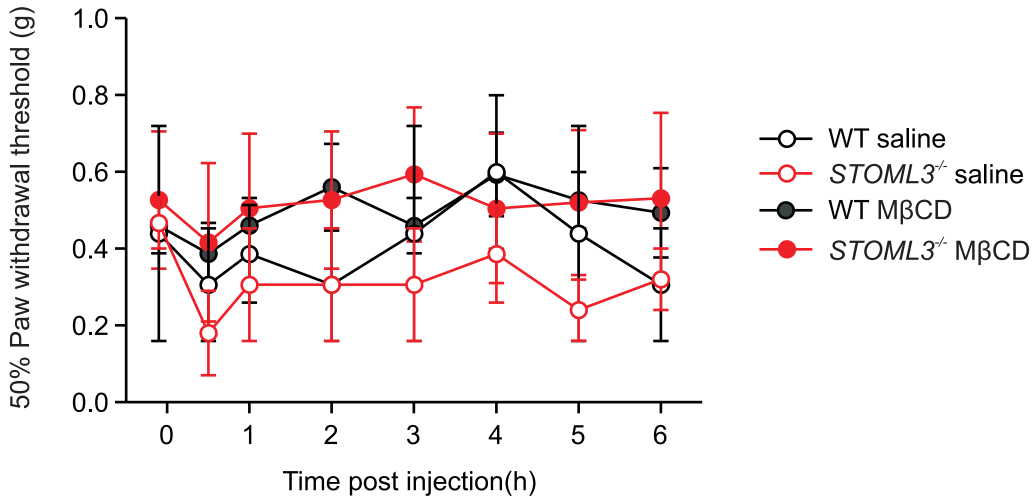

Supplementary Figure 9, Related to Figure 7

MβCD exhibited no effect on acute nociceptive response to mechanical stimulus in either C57BL/6N or *STOML3*$^{-/-}$ mice
(n=6 mice/group, MβCD effect: one-way ANOVA with post-hoc Dunnett's Multiple Comparison Test, C57BL/6N, $p>0.05$; *STOML3*$^{-/-}$, $p>0.05$; STOML3 influence: C57BL/6N versus *STOML3*$^{-/-}$, two-way repeated-measures ANOVA, $p>0.05$).

Supplementary Figure 9

Supplementary Table 1. Physiological properties of the mechanosensitive current

	WT Control n=58	WT MßCD n=60
**RA cells**	n=36	n=38
mean soma size (µm)	23.5±0.6 (17-35µm)	24.5±0.6 (19-32µm)
mean current amplitude (pA)	106.8±17.3	57.1±7.7**
latency (ms)	1.1±0.1	1.0±0.1
activation τ1 (ms)	0.51±0.06	0.38±0.05
inactivation τ2 (ms)	0.9±0.1	0.9±0.1
RMP(mV)	-64.2±0.8	-62.5±0.6
**SA cells**	n=13	n=3
mean soma size (µm)	22.1±1.02 (13-26µm)	25.0±1.4 (22-27µm)
mean current amplitude (pA)	107.2±20.7	141.0±104.0
latency (ms)	1.1±0.2	1.2±0.4
activation τ1 (ms)	0.52±0.08	0.55±0.26
RMP(mV)	-66.7±1.0	-63.3±2.6
**IA cells**	n=3	n=2
mean soma size (µm)	23.8±0.9 (22-26µm)	20.7±3.9 (16-25µm)
mean current amplitude (pA)	128.3±42.0	59.1±33.7
latency (ms)	0.9±0.1	0.3±0.8
activation τ1 (ms)	0.28±0.08	1.25±0.13
inactivation τ2 (ms)	14.1±3.6	13.5±7.3
RMP(mV)	-66.3±2.3	-66.5±3.5
**No response**	n=6	n=17
mean soma size(µm)	20.44±1.4 (18-27µm)	20.8±0.9 (16-28µm)
RMP(mV)	-60.67±2.1	-61.9±0.6

Physiological properties of cells recorded possessing an RA, SA or IA mechanosensitive current in control experiments and in experiments where cultures were treated with MβCD. For each group the mean resting membrane potential (RMP), cell soma diameter, mechanical latency, mechanosensitive current amplitude, and activation time constant or inactivation time constant is shown. No significant differences were noted between control neurons and the treatment groups, except for the marked decrease in the peak amplitude of RA current after MβCD treatment (unpaired t-test, $P<0.01$). **, $P<0.01$.

Supplementary Table 1

Supplementary Table S2. Values of the maximum current amplitude I_{max}, slope sensitivity *s* and activation stimulus midpoint $x_{1/2}$ of RA currents analyzed by fitting the current-displacement curves for control and MβCD treated groups to a Boltzmann distribution.

	Control	MβCD
	n=10	n=6
I_{max} (pA)	85.6±18.0	48.4±6.0*
s (pA/nm)	55.37±17.35	30.31±10.37*
$x_{1/2}$ (nm)	435.8±28.0	424.8±11.68

unpaired t-test, *, P<0.05

Supplementary Table 3. Values of the static tether force at zero F_0, the effective viscosity η_{eff} and the apparent surface tension T_{app} analyzed from the liner fit of tether force as function of pulling velocity.

	WT	*STOML3*$^{-/-}$
	n=20	n=19
$2\pi\eta_{eff}$ (pNsec/μm)	0.44±0.16	0.97±0.07
F_0 (pN)	30.9±1.6	26.2±0.7
T_{app} (pN/μm)	42±7%	31±3%

Supplementary Table 4. Membrane properties of sensory neurons and effects of cholesterol depletion

	1/2 AP duration <1ms		1/2 AP duration >=1ms	
	control	MβCD	control	MβCD
	n=44	n=49	n=33	n=23
soma size (μm)	24.3±0.4	24.6±0.4	23.2±0.5	22.7±0.9
RMP (mV)	-64.9±0.6	-62.6±0.5**	-65.7±0.7	-64.2±1.0
capacitance (pF)	43.7±3.6	36.0±3.0	34.4±3.5	29.5±3.6
resistance (MΩ)	44.9±4.6	39.1±3.9	89.2±20.7	84.4±8.4
AP threshold (nA)	1.87±0.15	1.60±0.12	1.47±0.16	1.39±0.12
1/2 AP duration (ms)	0.49±0.03	0.51±0.02	1.43±0.08	1.90±0.17*
AP amplitude (mV)	89.6±1.6	81.0±0.9	92.1±2.5	86.1±3.9

unpaired t test, *, P<0.05; **, P<0.01.
